# The spliced leader trans-splicing mechanism in different organisms: molecular details and possible biological roles

**DOI:** 10.3389/fgene.2013.00199

**Published:** 2013-10-11

**Authors:** Mainá Bitar, Mariana Boroni, Andréa M. Macedo, Carlos R. Machado, Glória R. Franco

**Affiliations:** Laboratório de Genética Bioquímica, Departamento de Bioquímica e Imunologia, Instituto de Ciências Biológicas, Universidade Federal de Minas GeraisBelo Horizonte, Brazil

**Keywords:** spliced-leader, trans-splicing, non-coding RNAs, RNA sequence analysis, RNA secondary structure

## Abstract

The spliced leader (SL) is a gene that generates a functional ncRNA that is composed of two regions: an intronic region of unknown function (SLi) and an exonic region (SLe), which is transferred to the 5′ end of independent transcripts yielding mature mRNAs, in a process known as spliced leader trans-splicing (SLTS). The best described function for SLTS is to solve polycistronic transcripts into monocistronic units, specifically in Trypanosomatids. In other metazoans, it is speculated that the SLe addition could lead to increased mRNA stability, differential recruitment of the translational machinery, modification of the 5′ region or a combination of these effects. Although important aspects of this mechanism have been revealed, several features remain to be elucidated. We have analyzed 157 SLe sequences from 148 species from seven phyla and found a high degree of conservation among the sequences of species from the same phylum, although no considerable similarity seems to exist between sequences of species from different phyla. When analyzing case studies, we found evidence that a given SLe will always be related to a given set of transcripts in different species from the same phylum, and therefore, different SLe sequences from the same species would regulate different sets of transcripts. In addition, we have observed distinct transcript categories to be preferential targets for the SLe addition in different phyla. This work sheds light into crucial and controversial aspects of the SLTS mechanism. It represents a comprehensive study concerning various species and different characteristics of this important post-transcriptional regulatory mechanism.

## Introduction

**Figure 1:**
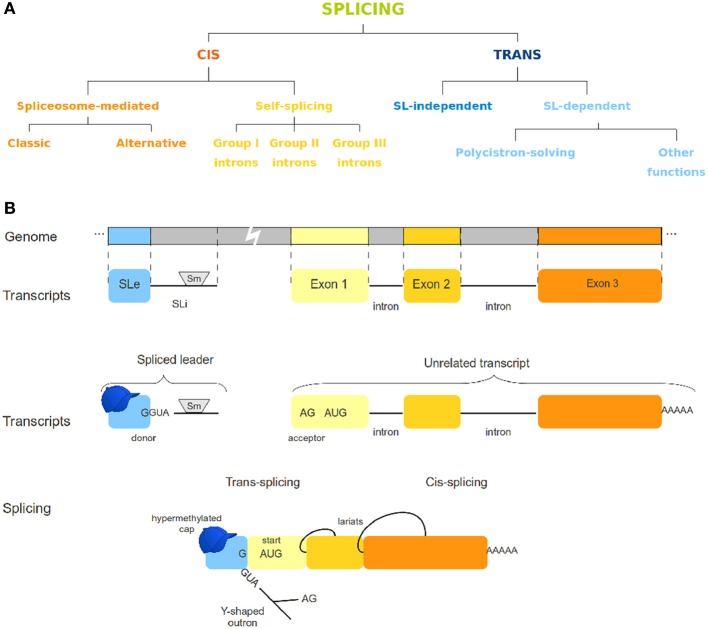
**Different splicing mechanisms. (A)** Variations of cis- and trans-splicing processes. **(B)** The spliced leader trans-splicing mechanism. This panel depicts the overall SLTS mechanism, in which an invariant exon (the SLe) is added to an unrelated, independently produced transcript, yielding a mature mRNA. Cis-splicing is also represented for comparison. The SLe molecule contains a hypermodified cap, whereas the SLi usually harbors an Sm-protein binding site.

Splicing has been known for a long time as a post-transcriptional process for regulation of gene expression [reviewed by Gilbert (1978); Padgett et al. (1986) and further by several other authors]. To date, there are different known variants of both cis and trans-splicing mechanisms (Figure 1A). Cis-splicing differs from trans-splicing on the genomic origin of the transcripts involved. While cis-splicing is concerned with transcripts from a single gene, trans-splicing mechanisms act by connecting transcripts of otherwise unrelated genes (Figure 1). In the spliced leader trans-splicing (SLTS) mechanism, the exonic portion of the spliced leader (SLe) transcript is transferred to the 5′ end of unrelated transcripts to yield a mature mRNA (Boothroyd and Cross, 1982) and reviewed by Liang et al. (2003) (Figure 1B). As first observed in trypanosomatids, its best described function is to resolve polycistronic transcripts into monocistronic units (Sather and Agabian, 1985) and reviewed by Preußer et al. (2012). Subsequently, SLTS was demonstrated to occur in other euglenozoans (Tessier et al., 1991) and several organisms, such as rotifers (Pouchkina-Stantcheva and Tunnacliffe, 2005), cnidarians (Stover and Steele, 2001), chordata (Vandenberghe et al., 2001), nematoda (Krause and Hirsh, 1987), platyhelminthes (Rajkovic et al., 1990), and dinoflagellates (Lidie and Van Dolah, 2007). Different biological roles have been proposed for this mechanism, such as: (i) enhancing translation of trans-spliced transcripts by providing a specialized (hypermethylated) 5′ cap structure for trans-spliced transcripts, (ii) stabilizing the mRNAs, and (iii) removing regulatory elements from the outron, what has been called 5′ UTR sanitization (Hastings, 2005; Matsumoto et al., 2010). In a recent work Nilsson and collaborators (Nilsson et al., 2010) discuss possible functions for the SLTS mechanism in *Trypanosoma brucei*. According to the authors, the differential insertion of the SLe sequence in alternative acceptor sites of genes could lead to: (i) translation blockage when the SLe insertion is upstream the protein start codon; (ii) alteration of protein subcellular location, when signaling sequences are eliminated by SLe insertion; (iii) inclusion or exclusion of uORFs or other regulatory elements from 5′ end of transcripts; and (iv) translation of alternative ORFs. It has also been speculated that SLe addition could lead to increased mRNA stability, differential recruitment of the translational machinery, modification of the 5′ UTR or a combination of those effects [reviewed by Hastings (2005); Stover et al. (2006); Lasda and Blumenthal (2011)]. From a recent study concerning the SLTS mechanism in the flatworm *Schistosoma mansoni* Mourão et al., (2013), our group identified transcripts under trans-splicing regulation in different life-cycle stages, suggesting that the SLTS could account for differential protein levels and protein repertories in different stages and/or environmental conditions. Although important aspects of the SLTS mechanism in this parasite were elucidated, several other questions regarding this mechanism in *S. mansoni* and other organisms remained unanswered. These questions surround the existence of conserved motifs within SL sequences from different species, the possible emergence of SLTS in more complex taxa as plants and mammals, the role of the SLTS mechanism in different organisms, the peculiarities of the set of transcripts under the control of a given SLe and the structural aspects of the SL molecule.

We performed analyses on a great number of SL sequences, searching for answers to such questions. All those answers lead to one final question, which has been debated in the literature for a long time: “what is the origin of the SLTS mechanism and how has it evolved?” This work is devoted to the proposition of hypotheses based on observed features of this mechanism that may guide the composition of a future final answer to this question. As for now, although there is no conclusive statement, there are important observations that can help the characterization of the mechanism, its biological role, phylogenetic features and molecular details. In the course of this study, we have compiled a comprehensive dataset of SL sequences from several species of various phyla. This was the starting point for several computational analyses that explored different aspects of the SLTS mechanism. To the best of our knowledge, no studies of this magnitude have been previously performed, regarding so many distinct features of this poorly understood mechanism in so many different species. Therefore, this work can largely contribute to a general overview of the SLTS in different biological contexts.

## Materials and methods

### Sequence retrieval and database generation

We performed manual searches in the National Center for Biotechnology Information (NCBI http://www.ncbi.nlm.nih.gov) database to identify previously annotated SLe sequences. The searches were guided by sequence features, especifically considering entries under the “miscellaneous RNA” sobriquet. The exact expression informed in the search field was “misc_RNA[Feature key]spliced leader.” Once sequences were retrieved, a manual curation was carefully performed to exclude false positives and reduce redundancy. A consistent preliminary database (hereafter named SEED database) was then compiled containing manually curated and annotated sequences. Notably, only sequences from species in which the SLTS mechanism was previously described were included.

Using the sequences from the former mentioned SEED database as queries, we performed searches using BLAST (Altschul et al., 1990) to expand the set of SLe sequences, and thereby generate a secondary dataset (hereafter named EXTENDED database). Searches were performed in the nucleotide collections (nt) database from NCBI using the blastn program (local version) with parameters automatically adjusted to address short sequences and allow for the retrieval of up to 500 matches for each query sequence. The results were then analyzed to identify SLe sequences based on information from the SEED database.

Several criteria were used to characterize a sequence as SLe to generate the EXTENDED dataset. As objective criteria, we only considered annotated sequences that displayed 90% or higher nucleotide identity and a query coverage exceeding 90% when compared to the respective query. More subjectively, for matches meeting the objective criteria, we have analyzed the presence of such sequences in the 5′ end of transcripts from these species. As an additional step, all uncharacterized sequences from organisms in which the presence of the SLTS mechanism was not previously demonstrated were not included in the datasets at first. Uncharacterized sequences were further analyzed to confirm or disprove those as SLe based on literature and sequence annotation.

Along with the retrieval of SLe sequences from the NCBI database, SL gene candidates were also retrieved to compose a separate database. SL gene candidates were identified based on sequence annotation and further analyzed for exclusion of false positives and redundancy reduction. Whenever duplicated sequences from a given species were identified, only one copy was kept to eliminate redundancy. After manual curation, the sequences were compared to the SLe from the same species present in the EXTENDED database and only candidates containing the entire SLe sequence followed by an intronic region were further considered to be SL genes.

### Sequence similarity assessment

During the construction of the previously mentioned databases, when more than one SLe sequence was retrieved for a given species, Clustal (Larkin et al., 2007) alignments were performed to guide redundancy reduction and false positive discovery. Subsequent manual alignments were performed to cluster sequences from the EXTENDED dataset. Sequence alignments were visually analyzed for the identification of duplicated sequences, such as completely identical sequences, partially identical sequences with missing residues and similar sequences presenting up to 5% (1 in every 20 positions, which is around the average size of the SLe sequences of most phyla) substitutions, deletions, or insertions. Notably, missing information at the 5′ end of SLe sequences is most likely to occur due to incomplete sequencing. For this reason, whenever data were missing for multiple 5′ end positions (more than 5%) in a sequence for a given species, conservation of such positions in the phylum was measured considering only the remaining sequences. To better observe consensus regions within SLe sequences, alignments were also used as input for the generation of sequence logos using the WebLogo suite (Crooks et al., 2004).

### Trans-spliced transcripts retrieval

Once the SLe EXTENDED database was built, its sequences were used as queries to search for transcripts from the respective species bearing the SLe in the 5′ end. BLAST searches were performed using the same parameters as described above, the same program (blastn) and reference database (nt). The results were manually curated to yield lists of transcripts that undergo SLTS in a given species. Those lists were joined together in a comprehensive set to allow further inspection of transcript conservation among species. All transcripts annotated as “hypothetical protein” or “unknown protein” were excluded from further analysis. Transcripts coding for identical proteins in different species were clustered together in a database to allow the assessment of conservation between species and across phyla of transcripts under SLTS regulation.

In parallel, data from the recently published work of Protasio et al. (2012) were used to yield a preliminary database of *S. mansoni* SLe-containing sequences. Fasta-format sequences for proteins coded by SLe-containing transcripts were retrieved from GeneDB (Logan-Klumpler et al., 2012), in which transcripts that undergo trans-splicing were associated with the gene ontology (GO) ID number 0000870. In both cases described above, after retrieval, GO terms were assigned to each transcript sequence using the GoAnna and GoSlim (McCarthy et al., 2006), tools from the AgBase web portal (McCarthy et al., 2010). Subsequently, manual annotation and classification according to the main biological function was performed for all transcripts (except those retrieved from the *S. mansoni* database) based on literature data.

### Secondary structure generation

To analyze structural conservation and topological features of the entire SL RNA molecule, all formerly mentioned SL gene sequences were submitted to RNAfold (Hofacker, 2003), a program from the Vienna package for RNA structure generation and energy assessment. Structures were then visually analyzed to search for conserved features in sequences from different species.

### Genomic location of SL sequences

The genomic location and copy number of the SLe sequences from two *Caenorhabditis* species were retrieved using BLAT (Kent, 2002) results and further analyzed. One hundred nucleotides downstream of each sequence were also retrieved to putatively represent the entire SL gene and allow for sequence comparison.

### One note regarding automatization

**Figure 2:**
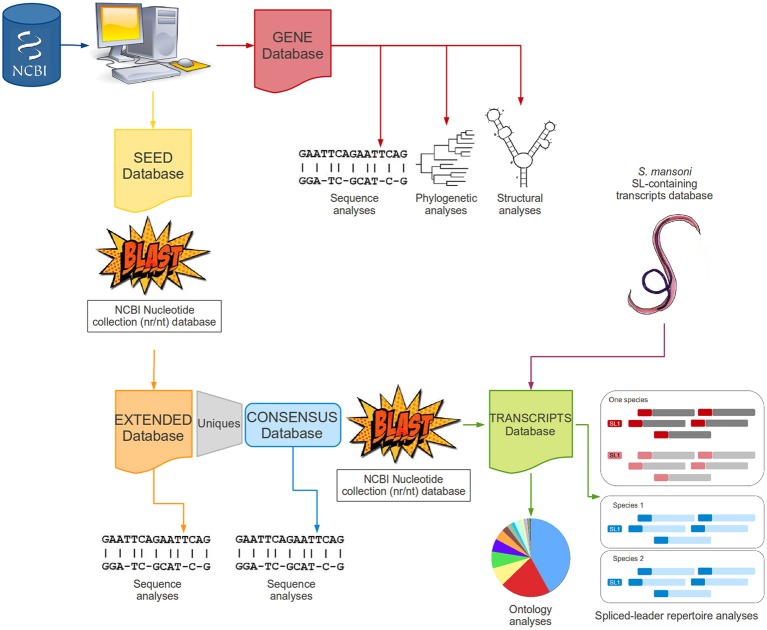
**Methodological workflow**. The figure schematically describes the main steps performed in this work as a fluxogram.

All steps were aided by simple *shell* and/or *perl* scripts to automatically manipulate files, organize and categorize data, recognize specific patterns within the text and perform searches in a file. A methodological workflow is presented in Figure 2 to illustrate each step of this study.

## Results

### Sequence retrieval and database generation

In the course of this work, we have assembled a comprehensive database of spliced leader sequences from several phyla. When a simple pattern-matching search was performed in NCBI's database to retrieve SLe sequences, 1161 matches were found. Among those, the majority (757) were from kinetoplastids and almost half (544) were specifically from *Trypanosoma spp*. The rest included sequences from nematodes (182), flatworms (98), dinoflagellates (95), cnidarians (4), rotifers (2), and chordates (1) phyla, in which the SLTS mechanism was previously described. Sequences from organisms of other phyla were also retrieved, but not included in the datasets because of the lack of previous consistent evidence supporting the presence of SLTS or because no transcripts were found bearing the sequence in the 5′ end. After redundancy reduction, false positive exclusion and validation by manual curation, a SEED database was defined containing only sequences with consistent evidence to be considered as SLe. This initial database was comprised 69 sequences from the seven different eukaryotic phyla (Supplementary Table 1): rotifera (2), chordata (1), cnidaria (2), dinoflagellate (8), euglenozoa (33), nematoda (18), and platyhelminthe (5).

These sequences were subsequently used as queries to extend the database based on BLAST similarity searches. The retrieved sequences were analyzed according to previously described criteria and a final EXTENDED database was generated that was comprised of 157 sequences from 148 different species (representing 81 genera) from the same seven phyla (Supplementary Table 2). Notably, all 157 sequences are, in fact, replicates of only 48 unique sequences, which were further clustered into 30 groups of highly similar sequences. This result indicates the high degree of sequence conservation, particularly between species from the same phylum (as will be further discussed). These 30 SLe sequences originated a third database which we named the CONSENSUS database (Table 1).

**Table 1:** **CONSENSUS Database**.

**Consensus name**	**Consensus sequence**	**Length (composition)**	**Plenty**
Rotifera 1	GGCTTATTACAACTTACCAAGAG	23 (8A/5C/4G/6T)	3/3
Chordata 1	GATTGGAGTATTTGGTTGTATTAAG	25 (6A/8G/11T)	7/7
Cnidaria 1	ACTTTTTAGTCCCTGTGTAATAAG	24 (6A/4C/4G/10T)	5/7
Cnidaria 2	CAAACTTCTATTTTCTTAATAAAG	24 (9A/4C/1G/10T)	2/7
Dinoflagellate 1	WCCGTAGCCATTTTGGCTCAAG	22 (4A/6C/5G/6T/1W)	23/23
Nematoda 1	GGTTTAATTACCCAAGTTTGAGGG	22 (6A/3C/5G/8T)	45/53
Nematoda 2	GGTTTAATTACCCAAGTTTAAG	22 (7A/3C/4G/8T)	2/53
Nematoda 3	GGTTTTAACCCAGTTAACCAAG	22 (7A/5C/4G/6T)	2/53
Nematoda 4	AGGTATTTACCAGATCTAAAAG	22 (9A/3C/4G/6T)	1/53
Nematoda 5	TACCGTTCAATTAATTTTGAAG	22 (7A/3C/3G/9T)	1/53
Nematoda 6	GTAATAAGAAAACTCAAATAAG	22 (13A/2C/3G/4T)	1/53
Nematoda 7	GGTTTTTACCCAGTATCTCAAG	22 (5A/5C/4G/8T)	1/53
Platyhelminthe 1	AACCGTCACGGTTTTACTCTTGTGATTTGTTGCATG	36 (6A/7C/8G/15T)	3/10
Platyhelminthe 2	AACCTTAACGGTTCTCTGCCCTGTATATTAGTGCATG	37 (8A/9C/7G/13T)	2/10
Platyhelminthe 3	AACTATAACGGYTCTCTGCCGTGTATATTAGTGCATG	37 (9A/7C/8G/12T/1Y)	2/10
Platyhelminthe 4	CACCGTTAATCGGTCCTTACCTTGCARTTTTGTATG	36 (6A/9C/6G/14T/1R)	3/10
Euglenozoa 1	AACTAACGCTATATAAGTATCAGTTTCTGTACTTTATTG	39 (12A/6C/5G/16T)	21/54
Euglenozoa 2	AACTAACGCTATTATTGATACAGTTTCTGTACTATATTG	39 (12A/6C/5G/16T)	12/54
Euglenozoa 3	AACTAACGCTATTATTAGAACAGTTTCTGTACTATATTG	39 (13A/6C/5G/15T)	4/54
Euglenozoa 4	AACTAAAGTTATTATTGATACAGTTTCTGTACTATATTG	39 (13A/4C/5G/17T)	2/54
Euglenozoa 5	AACTAAAGCTWTTATTAGAACAGTTTCTGTACTATATTG	39 (13A/5C/5G/15T/1W)	2/54
Euglenozoa 6	AACTAAAATTATTTATAATACAGTTTCTGTACTATATTG	39 (15A/4C/3G/17T)	1/54
Euglenozoa 7	AACTAAAGATTTTATTGTTACAGTTTCTGTACTATATTG	39 (12A/4C/5G/18T)	1/54
Euglenozoa 8	AACTTACGCTATAAAAGTCACAGTTTCTGTACTTTATTG	39 (12A/7C/5G/15T)	2/54
Euglenozoa 9	AACTAACGCTATTATTGTTACAGTTTCTGTACTTTATTG	39 (10A/6C/5G/18T)	3/54
Euglenozoa 10	AACTAACGCTAWAAAAGWTACAGTTTCTGTACTTTATTG	39 (13A/6C/5G/13T/2W)	2/54
Euglenozoa 11	AACTAACGCATTTTTTGTTACAGTTTCTGTACTTTATTG	39 (9A/6C/5G/19T)	1/54
Euglenozoa 12	AACTAACGCTATATTTGTTACAGTTTCTGTACTWTATTG	39 (10A/6C/5G/17T/1W)	1/54
Euglenozoa 13	AACTAACGCTATTCTAGATACAGTTTCTGTACTTTATTG	39 (11A/7C/5G/16T)	1/54
Euglenozoa 14	AACCAACGATTTAAAAGCTACAGTTTCTGTACTTTATTG	39 (13A/7C/5G/14T)	1/54

^*^Total number of sequences in the phylum/Number of sequences matching consensus.

In addition to the phyla previously represented within the SEED database, sequences from other phyla have also met the requirements to be considered SLe but were not included in the EXTENDED database. Species of arthropods, ciliophora, echinodermata, mollusca, mycetozoa, apicomplexa, plants and even a bacterial species presented putative SLe sequences (Supplementary Table 3), although most of these phyla have never been proven to harbor the SLTS mechanism. No obvious decision on whether they are real SLe sequences could be reached, and those were therefore excluded from our dataset.

### Description of the EXTENDED database

**Figure 3:**
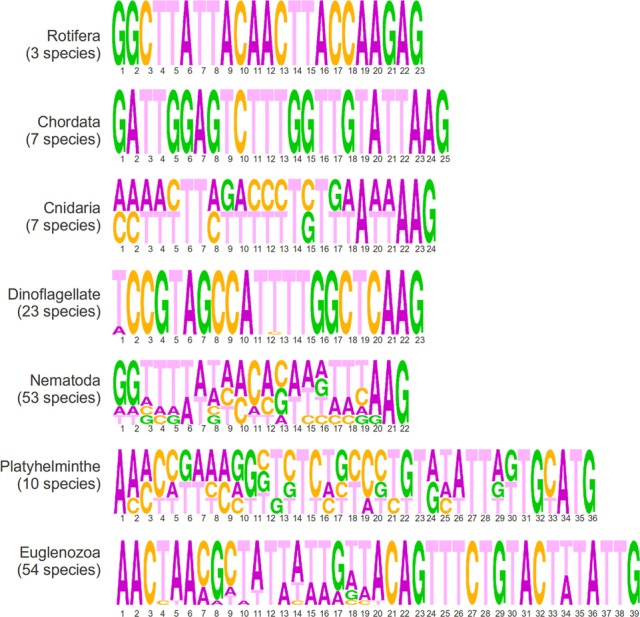
**Sequence logos generated from the alignment of all SLe sequences from each phylum showing sequence lengths in numbers and the frequency of the four nucleotides in each position**.

In this section, we present a short description of sequences in the EXTENDED database according to phyla, which is fully available as a supplementary file (Supplementary Table 2) and graphically summarized in Figure 3. There are three species of rotifera in the dataset that share an identical 23 nucleotide SLe sequence that is enriched in adenines. All seven chordata species share an identical SLe sequence, apart from missing residues. The consensus is a 25 nucleotide sequence enriched in thymine nucleotides. Cnidarians are also represented by seven sequences from five species of the same genus (*Hydra*). All species share the exact same SLe sequence and, among those, two of the species present an additional SLe sequence, which is identical in both species. The two consensus sequences are composed of 24 nucleotides, of which, 10 are thymines. All 23 species of dinoflagellates in the database share an identical 22 nucleotide SLe sequence that is only degenerated in the first position (either A or T), with a balanced nucleotide composition. Notably, this is the only phylum in which, the SLe sequence itself carries the Sm-protein binding site (a T-rich element, which like in *C. elegans* and many other species is a AT_4−6_G motif). There are 53 sequences from the nematoda phylum within the database. Among these, 45 are identical apart from a repetition of the nucleotide G of variable lengths at the 3′ end. Among the remaining eight sequences, two are identical pairs and four are unique, one of which harbors an Sm binding site (the other two sequences present a putative inverted Sm binding motif). Most sequences have 22 nucleotides (apart from the variable repetition of guanines), and the majority are slightly richer in A and T nucleotides. Among the platyhelminthes, there are 10 species from seven different genera represented in the dataset. All SLe have a high percentage of thymines and are 36 or 37 nucleotides long. The sequences can be divided into four different groups of two or three virtually identical sequences each. Notably, each group contains species of a unique order. A classical Sm-protein binding site was found in one of such groups, and another group presents a putative inverted binding site. Euglenozoans are unique organisms in this context because in these species all transcripts are processed by SLTS. This phylum is represented by 54 sequences of 39 nucleotides from 14 different genera. The sequences can be clustered together in 8 groups of identical sequences plus 6 isolated sequences that are not identical to any other sequence. In summary, the terminal regions (first 6 and last 20 nucleotides) are conserved in all sequences, whereas the central region is variable. All 16 *Leishmania* species share the exact same sequence, whereas the 22 *Trypanosoma* species were divided into 4 groups with identical sequences and 2 isolated sequences. Alternatively, with less stringency, SLe from this phyla could be clustered into three groups according to specific signatures within the last 20 nucleotides: (i) *Trypanosoma* species; (ii) *Leishmania, Leptomonas, Wallaceina*, and *Chritidia* species (with identical sequences); and (iii) the remaining genus, which presents more diverse sequences (the data presented in this section are shown in Figure 3, Table 1 and Supplementary Tables).

### SLe sequence comparison among phyla

When analyzing the EXTENDED dataset of SLe sequences, a high sequence conservation within each phyla was revealed, but a very low conservation among different phyla was found. One interesting feature we highlight as a general tendency is that sequence length is more conserved than sequence composition itself in any given phyla. There are some clear differences between sequences from euglenozoa and platyhelminthes and those from other phyla. Such differences include the sequence length and terminal residue identity. Whereas sequences from all other phyla range from 22 to 25 nucleotides in length, sequences from euglenozoans and platyhelminthes are 39 and 36–37 nucleotides long, respectively. As for the nucleotides at the 3′ end, in which SL exon-intron cleavage occurs and the SLe is incorporated into mRNAs, euglenozoans and platyhelminthes have TTG and ATG patterns, respectively, whereas sequences from other phyla have an AAG pattern (except for one nematoda consensus sequence and the sequences from rotifera that end in GAG). Regarding the 5′ end, 17 of the 18 consensus SLe sequences from euglenozoa and platyhelminthe present a conserved AAC pattern as the first nucleotide triad. For all other phyla, there is no conservation of nucleotides in the 5′ end. Notably, all but five sequences from the CONSENSUS dataset present a TTT triplet, which in all dinoflagellates and three species from the other phyla, are part of the Sm binding site (in five other species it is part of a hypothetical inverted Sm binding motif) (Table 1). When considering all sequences in the CONSENSUS database, there is an evident enrichment in adenine (~30% of all nucleotides) and in thymine (~40% of all nucleotides) in comparison to guanine and cytosine (which together comprise only ~30% of all nucleotides).

### SL gene sequence comparison among all phyla

Several candidate SL gene sequences were identified through manually analyzing the retrieved sequences from the NCBI database. Within the preliminary dataset, we performed a manual curation to reduce redundancy and exclude false positives, and we also mapped the available SLe sequence to the initial portion of the putative genes of the same species. As a result, 30 sequences remained and were further separated according to phyla and analyzed. Gene length was relatively variable, from 75 to 123 nucleotides; although most sequences were approximately 100 nucleotides long (mean length is 107). The most evident patterns within sequences are the cleavage site in the exon-intron boarder and the presence of the Sm-protein binding site, which is usually in the intronic portion of the gene. Nevertheless, there is some degree of SLi sequence conservation among species from a given phylum, although it is much lower than for the SLe sequence alone. Differing from the exonic portion, the intronic nucleotide composition has an almost equal distribution of nucleotides with 24% adenine, 24% cytosine, 26% guanine, and 26% thymine.

### SL genomic location and composition in two *caenorhabditis* species

Regarding the genomic position, it is already known that most SL genes are located near the 5S ribosomal RNA gene and comprises multiple copies in tandem (apud Hastings, 2005). We have used BLAT to map SLe sequences in the genomes of *C. elegans* and *C. remanei*. Both species have two different SLe sequences in the database, and these sequences are identical between species. One sequence [hereafter named as SLeI and identical to the SL1 sequence from Ross et al. (1995)] is shared among the majority of the nematoda species (45 of the 53 sequences from this phylum), and the other [hereafter named SLeII, closely related to the SL2 first described by Huang and Hirsh (1989) and identical to SLf as described by Ross et al. (1995)] is only common to these two *Caenorhabditis* species in our database.

In *C. elegans*, the SLeI sequence is repeated 13 times in chromosome V, twice in chromosome I and once in chromosome III (thus totaling 16 copies). The SLeII sequence is repeated twice in chromosome I and has only one copy in chromosomes III and IV (thus totaling four copies). The genome of *C. remanei* is more highly populated by SLeI sequences, which are represented by 135 copies. The SLeII sequence on the other hand seems to have only nine copies. The karyotype is not available in BLAT for the later species and therefore, it was not possible to map sequences onto chromosomes. Notably, the SLeI sequence, which is widespread among nematoda species, is always more represented in the genomes of these two species (16 vs. 4 copies in *C. elegans* and 135 vs. 9 copies in *C. remanei*).

Regarding the intronic portion of the putative SL gene sequences, we have analyzed all occurrences of the *C. elegans* SLeI in chromosomes V, III, and I. We observed that 10 repetitions have identical introns (all in chromosome V) and that the other putative SLeI gene sequences are very divergent from one another. Notably, these 10 identical sequences are the only ones to present classical Sm-protein binding site. When evaluating the intronic portion of the four putative genes with the SLeII sequence in *C. elegans*, a high similarity is observed, although the sequences are not identical. These sequences also present Sm binding sites, although three of these have five thymine within the repetition, whereas the additional sequence has four. We have noticed a conserved GTTAG pattern in the four putative SLeII gene sequences that is also present at a different position in the 10 identical sequences and is absent in all other six putative SLeI gene sequences (two other short patterns—ACAA and GGAA—are also present in the 14 sequences, but are not exclusive to these sequences). Among the nine sequences of the putative SLeII genes in *C. remanei*, eight are identical (apart from one substitution in two sequences) and the other contains approximately 10% substitutions. All nine sequences contain classical Sm-protein binding sites. In addition, these sequences are closely related to the putative SLeII gene sequences of *C. elegans*, although not identical. When analyzing the set of 135 putative SLeII gene sequences in *C. remanei*, several clusters of highly similar or identical sequences are observed. One important observation is the lack of Sm binding sites in most of the sequences. Only 27 sequences bear Sm binding site motifs. These can be divided into groups of identical or nearly identical sequence; one of which is also closely related to the main group of *C. elegans* putative SLeI gene sequences. We have reconstructed a phylogenetic tree with all the SL sequences from both species (data not shown) and observed a more randomized distribution of SL genes not bearing Sm binding sites in comparison to sequences that contain this motif. We then narrowed our analysis to consider only Sm binding-containing sequences, and the corresponding tree is shown in the supplementary material (Supplementary Figure 1). The tree is divided into three main groups: (i) a group of SLeI gene sequences containing all *C. elegans* and approximately half the *C. remanei* SLeI gene sequences, (ii) a group of all SLeII gene sequences and (iii) a more distant group of *C. remainei* SLeI gene sequences. Each group can be further clustered into smaller groups with closely related sequences.

### SL gene structural comparison

**Figure 4:**
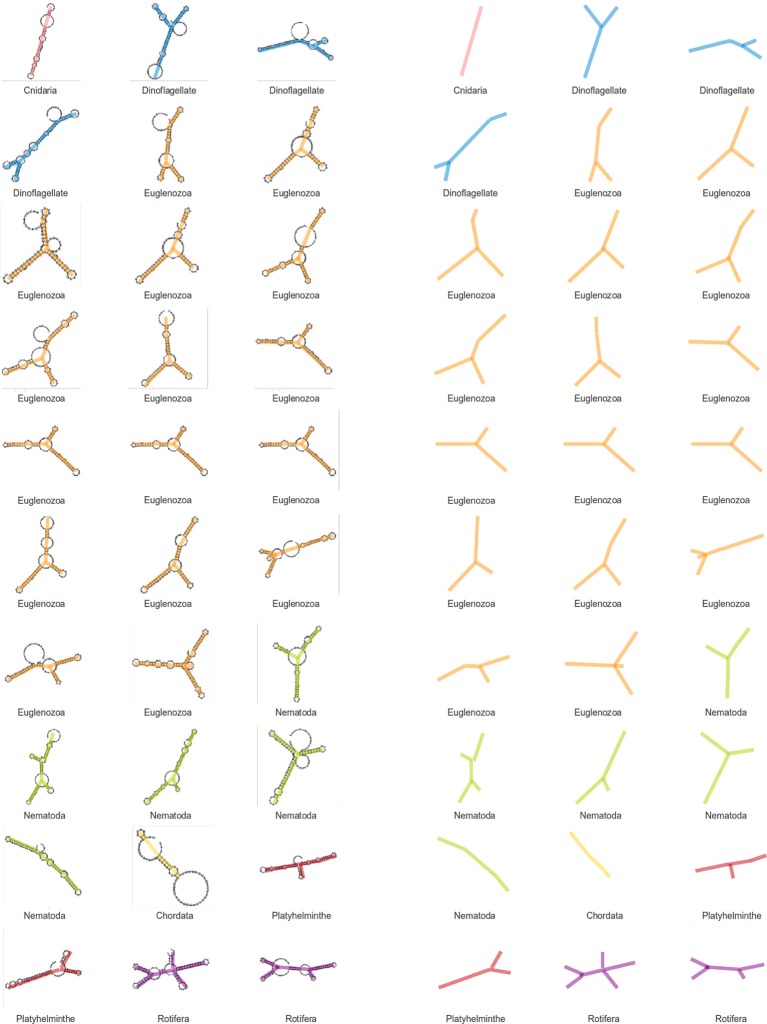
**Structures of SL genes**. Secondary structures were generated for SL genes of different phyla and a visual analysis of these structures point to a conserved Y-shape for the SL molecule. The backbones of the structures are displayed to the right for a simpler view.

We have assigned secondary structures to all 30 SL gene sequences with RNAfold from the Vienna package. As a result of this structural analysis, a tendency for SL RNAs to form a Y shaped molecule was observed (Figure 4). The topology is the result of three stem-loops and a branch point and seems to be conserved in nearly all analyzed species, although the branch point position and stem-loop length are variable. The average free energy (ΔG) value for gene structures in fixed secondary structures was −30.6 Kcal/mol (ranging from −14.4 to −51.80 Kcal/mol).

### Analysis of transcripts trans-spliced to specific SLe

To assess whether the sets of trans-spliced transcripts from different organisms harboring the same SLe sequence are similar, we conducted BLAST searches in the nr/nt database using two different sequences as queries: (i) a sequence shared by different dinoflagellate species and (ii) a sequence shared by different nematoda species (other than *C. elegans*). For each SLe, we have allowed retrieval of up to 500 transcripts from different species of each phylum (dinoflagellate and nematoda). We have excluded *C. elegans* from our survey because in this organism the addition of different SLe sequences in different transcripts has been investigated by high throughput sequencing (Allen et al., 2011) and could not be included here without introducing a substantial bias to the analyzed data. Among all retrieved dinoflagellate sequences, 133 remained after false positive exclusion and redundancy reduction. From these, 63 (over 47%) transcripts were shared by more than one species (totaling 30 different transcripts, from which many code for ribosomal proteins). The remaining 70 are specific to only one species. From the nematoda transcripts, after manual curation, we analyzed 158 transcripts from which 54 (over 34%) are shared by different species. We then decided to analyze whether in one given species two different SLe sequences would regulate different sets of transcripts. To this end, we used BLAST and searched the nr/nt database for transcripts from *H. vulgaris* bearing any of the two different SLe sequences. As a result we have identified 30 transcripts trans-spliced with one sequence and 13 with the other, none of which were related to both SLe sequences.

### Analyses of trans-spliced transcripts from all species

**Figure 5:**
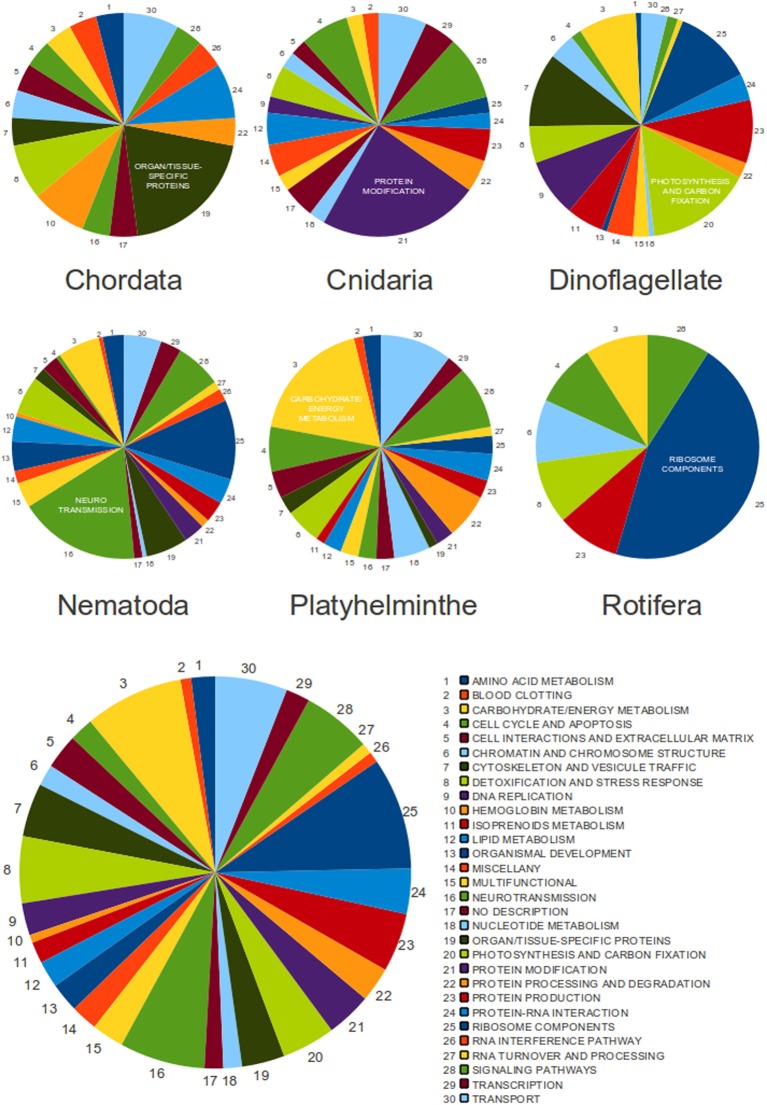
**The classification of SLe-containing transcripts from all analyzed species**. Pie charts are presented for each phylum individually (upper charts) and for the entire set of 455 transcripts (bottom chart). Functional classes are represented by color, and the arrow indicates the direction that corresponds to the legend. The most populated class for each phylum is labeled by name.

In a similar context, a more overall analysis was performed with the unique sequences of the EXTENDED database of SLe sequences as queries for BLAST searches (with the blastn program) within the nr/nt database. Search results and manual curation resulted in a final set of 455 transcripts (Figure 5 and Supplementary Table 4), among which five were annotated as “alternatively spliced.” From this set, we have previously excluded transcripts from euglenozoa species because in these organisms, SLe addition is ubiquitously used to solve polycistronic transcripts. Among these 455 transcripts, 237 are present in only one species and 218 are shared by more than one species (totaling 60 unique sequences). Additionally, 138 are common to species from different phyla (totaling 32 unique sequences). Enolase, calmodulin, gluthatione S-transferase, ATP synthase subunits, cyclins, eukaryotic translation initiation factors, superoxide dismutase, ras-related proteins, and ribosomal proteins are among the most ubiquitous trans-spliced proteins, as these are found in species of at least three different phyla.

We have automatically assigned GoSlim terms to all transcripts and the results revealed no clear bias to any specific gene category. This was true for the entire set of transcripts and also for each individual phylum (data not shown). Because this protocol was not curated and has classified transcripts into generic and less informative classes, we have decided to manually annotate and classify all transcripts aiming to achieve a more specific, reliable and informative result. We have therefore separated transcripts according to their main biological function, generating a total of 30 different functional classes with different representations. The result was unexpected and revealed unique classes composed of transcripts that seem to more frequently undergo trans-spliced in each phylum, although this was not true for the entire set of transcripts, in which no specific category seemed to be dominant (Figure 5 and Supplementary Tables 4, 5).

In parallel, using data available from the work of Protasio et al. (2012), we have generated a set of 1411 SLe-containing transcripts from *S. mansoni*. Preliminary analyses after GO assignment and GoSlim retrieval revealed no clear bias regarding the biological processes under the control of the SLTS mechanism, although the number of transcripts in a few biological processes was higher, such as whole-cell functions, metabolic processes, and organismal development (data not shown).

## Discussion

### Are SLe sequences conserved among different species?

One of the most discussed topics related to the SLTS mechanism is its evolutionary origin. There is previous evidence for a unique origin in a common ancestor but there is also evidence for multiple unrelated origins (Nilsen, 2001; Stover and Steele, 2001; Hastings, 2005). From the 157 sequences comprising the described EXTENDED database, it is possible to observe few features that could universally define a SLe. Almost all sequences have a conserved WWG at the 3′ end and a TTT pattern. As for the latter, we suggest it may be the vestige of a Sm binding site motif that was once present within the SLe and was further lost or transferred to the intronic portion. Notably, there is a high sequence conservation rate within phyla, specifically to the level of subfamilies, although only the two aforementioned features are conserved among unrelated phyla. In addition to the sequence itself, the SLe length is even more conserved within phyla, indicating this as a crucial characteristic of the molecule.

There are important characteristics that differentiate euglenozoa and platyhelminthe consensus SLe sequences from other sequences, such as the length and composition of the 5′ and 3′ ends. This could reflect a distinct role of the SLTS mechanism in such species. Regarding trypanosomatids, the SLTS mechanism has a crucial role because in these organisms large regions of the genome are transcribed as polycistronic units and SLe incorporation is crucial for their resolution into monocistronic units. In platyhelminthes, the mechanism is supposed to be acting as an expression regulator for approximately 10% of all genes. A unique feature of the SLe sequence in this phylum is the presence of an ATG as the last nucleotide triad. This could account for an intrinsic start codon, that could generate an alternative open reading frame (ORF) in which the SLe insertion occurs within the transcript sequence, thus giving rise to alternative forms of the resulting protein. Nevertheless, the relationship between sequences from platyhelminthes and euglenozoans is not clear nor it is phylogenetically expected because these two phyla come from different eukaryotic kingdoms (animalia and excavata, respectively).

### Are SL gene sequences conserved among different phyla?

Similarity between SL gene sequences from different phyla is less evident, but some features are conserved. Specifically, the last nucleotide of all SLe sequences is a guanine (G). This is because the cleavage site between the exon and intron is a highly conserved GGTA motif, from which the first nucleotide (G) is the last nucleotide of the exon and the other three (GTA) are in the beginning of the intron. Another conserved motif is the binding site for the Sm protein (usually a AT_4−6_G motif). This conserved binding site is usually present in the intron, with the exception of the dinoflagellate sequences (and a few sequences from other phyla), in which the Sm binding site is located in the exon. The consequences of this exonic localization of the Sm binding site are not completely understood. Notably, intronic nucleotide composition is homogeneous (not biased for a specific nucleotide type), indicating a tendency for a lack of selective pressure in this region, except for the presence of the Sm binding site. From all analyzed phyla, euglenozoa species present the longest SLe sequences, with 1.5 times the size as compared to SLe from other species (which average 23 nucleotides). This discrepancy may indicate an independent origin of SLTS in euglenozoa, which may in turn be related to the unique transcription strategy adopted by such organisms and the role of SLe insertion in the post-transcriptional processing of polycistronic transcripts.

### How conserved are putative SL genes in species of *caenorhabditis?*

There are two different SLe sequences in each of the two *Caenorhabditis* species in the EXTENDED dataset (we have internally named these as SLeI and SLeII). These sequences were mapped with BLAT on the genomes of the respective organisms, and the retrieved sequences of the putative SL genes (the SLe sequence plus 100 nucleotides downstream) were analyzed. The results show a differential abundance of each SLe in the genomes, with the SLeI sequence being the most abundant in both species. Notably, this SLe is shared with the majority of the nematode species represented in the database. In each species, regarding sequence similarity, putative genes of identical SLes seem to be more related to one another than to putative genes of other SLe. Although not all sequences are identical for a given SLe, sequence alignments present groups of closely related introns.

In their 1987 article, Krause and Hirsh (1987) reported the existence of more than 100 SLeI genes in *C. elegans*, which is much higher than we have observed. This discrepancy can be explained by the methodology used for SL gene identification, which considered sequences that were a 90% match to the SLe (20 out of 22 nucleotides) and was performed by Southern blot analysis. By contrast, in this study, we considered only 100% matches in the genome, thus restricting the number of retrieved sequences. Unfortunately, because the *C. elegans* genome was not available in 1987, we cannot compare our results to the previously published results.

When analyzing putative intronic sequences of different SLe, sequence conservation is more clear among species. There are only 10 SLeI gene sequences in *C. elegans* bearing the Sm binding site, and these are closely related to one group of SLeI sequences of *C. remanei*, in which only 27 sequences contain Sm binding sites. All of the remaining sequences (not bearing the Sm binding site) do not seem to be related to one another. The scenario is simpler for the SLeII sequences because there are fewer in both species. All four *C. elegans* SLeII sequences contain Sm binding sites and are similar. The nine *C. remanei* SLeII sequences are nearly identical, contain Sm binding sites and are closely related to the *C. elegans* SLeII sequences.

When we analyze the phylogenetic tree in the supplementary material (Supplementary Figure 1), we see two main groups: one comprising approximately half *C. remanei* SLeI gene sequences and another containing all other sequences. The latter is further divided into two groups, one with the SLeII gene sequences and another with SLeI gene sequences. This seems to indicate the existence of a more ancient SLe from which the SLeI and SLeII genes have arisen by duplication, most likely before speciation between *C. elegans* and *C. remanei* (given the similarity among intronic sequences of both species). The second group of SLeI genes from the later species may have arisen by duplication after speciation or (most likely given the divergence among sequences in this group) may be a result of duplication from a common ancestor prior to speciation and was then lost by the former species. Taken together, these results show that the orthologous SLe gene sequences (identical SLes in different species) are more related to one another than to paralogous genes (other SLe in identical species). This most likely indicates that divergence between SLeI and SLeII took place before speciation.

### Are different SLe sequences from one unique species incorporated into different transcripts?

This study observed that a given species may have more than one SLe sequence, supporting the literature [as in the classic case of *C. elegans* first reported in Huang and Hirsh (1989)]. This could account for the differential expression of transcripts or the production of different protein repertories under certain environmental conditions or developmental stages. We have identified two different SLe sequences from *Hydra vulgaris* and analyzed the set of transcripts related to each in public databases. Although this cannot be considered a fully conclusive analysis, it gives an indication of the possible roles for different SLe sequences. Considering the two distinct SLe from *H. vulgaris*, whereas one was found in 30 annotated transcripts, the other was found in less than half of these (only 13 transcripts). There was no superposition of the two sub-groups, indicating that each SLe sequence could be trans-spliced to a distinct set of transcripts. Notably, the SLe that was added to a higher number of transcripts is the most conserved when compared to other species from identical genera. This result is in agreement with the recent work of Allen et al. (2011), where the authors conclude that in *C. elegans* the trans-splicing to SLeI or SLeII are mechanistically separate and distinct phenomena. On the other hand, the fact that in the consulted database both SLe sequences were related to the same number of transcripts is not expected given the higher prevalence (of >80%) of SLeI trans-splicing in *C. elegans* as reported on Allen et al. (2011).

### Are identical SLe sequences in different species incorporated into different transcripts?

The analysis of transcripts bearing SLe sequences that are shared by different organisms from a same phylum indicated that these SLe sequences may control similar transcript repertories. The first SLe sequence that was analyzed is the one conserved in all dinoflagellate species in the database. As a result, from the retrieved matches, 133 transcripts remained after curation with almost half (63) shared by more than one species. The other analyzed SLe is common to several nematode species. In this case, from the retrieved matches, 158 transcripts remained after manual curation, 54 of which are shared by different species from the same phylum. In the latter case, we have excluded *C. elegans* from our search, since in this organism it is already known that SLeI and SLeII have different roles and are therefore added to different sets of transcripts (Allen et al., 2011). Taken together, these results may indicate that identical SLes in different species from the same phylum are incorporated into the same transcripts. The observation that not all transcripts are common to all species harboring identical SLe sequences could be a result of the restricted number of annotated transcripts deposited in publicly available databases. Sets of transcripts from different species that are regulated by the same SLes, have between 34 and 47% common elements, potentially indicating an overall tendency for each SLe to regulate specific transcripts, regardless of the species. If this is true, then a given SLe will always be related to a given set of transcripts in all species (except maybe for species-specific genes), and therefore, different SLe from identical species would regulate different sets of transcripts. Indeed, Allen and collaborators have shown that, in *C. elegans*, spliced leader sequence SLeI is added either to monocistronic genes or to the first gene of a polycistron, while the SLeII sequence is added to internal genes of a polycistron. These two trans-splicing events are therefore mechanically unrelated and may be functionally different. This observation alone could account for the lack of overlap between the sets of transcripts regulated by each SLe sequence in this species and maybe other species that carry genes in an operon structure. Additionally, the authors show that some genes can actually be trans-spliced to both SLeI and SLeII and these are internal genes in the polycistron for which there are independent promoters. Results show that, for genes preferably trans-spliced to SLeII sequences, some level of SLeI addition may still be observed. According to the authors, this may be due to the 10-fold excess of SLeI in the cells in comparison to SLeII or it may be that SLeI is added to transcripts in a constitutive frequency, while SLeII addition could be more specific. Unfortunately, there are limited data on trans-spliced transcripts of other species with multiple SLe sequences and therefore it was not possible here to reproduce the same observation in other species, apart from a limited analysis of *H. vulgaris* transcripts, in which we have found 30 SLeI-containing transcripts and 13 SLeII-containing transcripts, with no overlap among these sets.

### Are transcripts regulated by the trans-splicing mechanism common to different species and phyla?

We have analyzed over 450 transcripts bearing SLe sequences from all species in our EXTENDED database except euglenozoans (because this species add SLe to all transcripts). Of those species, almost half (48%) contain transcripts shared by at least two different species and almost one third (30%) contain transcripts of species from different phyla (136 transcripts, representing 32 unique sequences). Among the 32 transcripts represented in multiple phyla, most are conserved in all eukaryotes, and the related proteins perform basic functions in the cell, including involvement in ribosomal activity, cell structure, ATP synthesis, glucose metabolism, protein folding, antioxidant defense, DNA replication and translation. This may reflect a tendency of the SLTS mechanism to regulate ancient and conserved functions.

To thoroughly investigate the relationship between trans-spliced transcripts from different species and phyla, we have manually annotated and classified all 455 transcripts according to their biological function. In a surprising result, we have identified a dominant class of transcripts in each phylum, with no overlap among phyla. This could indicate that each phylum may have a preference to add the SLe to a specific gene category. We did not perform analyses to investigate if this is true at the species level, although previous data from studies in *S. mansoni* suggests it is not true (Mourão et al., 2013 and our previously described GO annotation of SLe-containing *S. mansoni* transcripts). There is no overall tendency when we observe the set of transcripts from all phyla. This is expected because each phylum has a bias to a different class. Notably, we have not identified genes related to host-parasite interactions undergoing SLTS in the parasitic organisms. We consider this to be an expected observation if one considers that the SLTS mechanism was derived early in the eukaryotic lineage. On the other hand, photosynthesis is the major class of trans-spliced transcripts for the dinoflagellate species (from which only two *Perkinsus* species are not photosynthetic) and this is a phylum-specific class in our study because no species out of this phylum perform photosynthesis.

We cannot confirm that the observed bias to specific functional categories is not expected for a given phylum because we did not perform the same annotation and classification protocol for the entire set of transcripts for each species. For example, it is reasonable to expect that energy metabolism would be a broadly represented class in the transcriptome of most organisms and, accordingly, it is the major class among platyhelminthe transcripts that undergo trans-splicing. However, in nematodes there is a bias for transcripts involved with neurotransmission to undergo trans-splicing. This is not an expected result. Despite the evidence above, there remains a limited number of SLe-containing transcripts in public databases, therefore, no final conclusions can be reached.

### Are the transcripts undergoing trans-splicing in a given organism related to one another?

As we have observed in a previous study regarding the SLTS mechanism in *S. mansoni* (Mourão et al., 2013), we found no bias for any specific functional gene category to have transcripts undergoing SLTS. In a preliminary survey using a recently published *S. mansoni* SLe-containing cDNA dataset (Protasio et al., 2012), we have used GO annotations to assess whether specific gene categories could be found among the 1411 SLe-containing transcripts. The most abundant biological processes are whole-cell processes (as cell differentiation, cell cycle, cell death, cell communication, cell proliferation, cell growth, cell recognition, and cellular homeostasis), cellular component organization, transport, response to stimulus, signaling, protein function, protein expression, metabolic processes and organismal development, notably multicellular organismal development (which is the class with the highest number of related transcripts).

Taken these observations together, we can conclude that the SLTS mechanism does not regulate any specific biological process category. Nevertheless, some categories are slightly more represented than others and those categories are crucial processes for the metabolism of the cell and the organism. We can then speculate that the SLTS mechanism is of fundamental importance and that the disruption the mechanism should lead to serious consequences for the organism. This represents a notable result that places the SLTS mechanism in an important place for organismal development and survival.

### Are SL RNA structures conserved among different sequences?

A structural RNA analysis was performed to identify possible structural conservation despite the lack of sequence similarity across phyla. Structures were generated for the 30 SL gene sequences mentioned previously, and the results do not show a clear conservation, although some features may be observed. Because SL gene sequences vary in length, structure complexity is also diverse. An almost universal topological identity can be observed among the different SL structures, with few exceptions. SL RNA structures have three stem loops and a bifurcation point. This topology can be defined as a Y shaped structure. Although the bifurcation point location and stem-loop length may vary, the overall topology may be the only common aspect providing the SL RNA structure, which is a feature that is shared by all species.

## Conclusions

In the course of this study, we have investigated several characteristics of the SLTS mechanism in different species from various phyla. Analyses of the conserved features revealed a close relationship between SLe sequences from the same phylum. However, sequences from different phyla do not share many common features. SL structures show a certain level of topological conservation across phyla with an overall Y-shaped structure.

Transcripts controlled by the SLTS mechanism are, to a certain extent, shared among different species and organisms from different phyla, although different biological functions are the focus of SLTS in different phyla. Keeling et al. (2005) have published a consistent classification for eukaryotes that includes five different “supergroups,” namely excavates, rhizaria, unikonts, chromalveolates, and plantae. Those phyla in which the SLTS mechanism was previously characterized are located in three of such supergroups: unikonts (rotifera, chordata, cnidaria, nematode, and platyhelminthe), excavates (euglenozoa), and chromalveolates (dinoflagellates). The widespread presence of the mechanism could place a unique origin in an ancestor common to all eukaryotes (or at least to these groups). The outcome of this hypothesis, if supported, is the previous existence of the SLTS mechanism in all eukaryotes, although the progressive loss of relative importance reduces the chance of identifying SLe-containing transcripts in more complex organisms. It may even be true that some species have completely lost the SLTS mechanism when more robust regulatory mechanisms emerged. Despite speculation, no final answer can be reached and this is partly due to the restricted data currently available. Although we have covered here all species in which SLe sequences are annotated and deposited at NCBI, more data (specifically of species from other phyla) is needed to more precisely place the origin of this mechanism among eukaryotes.

Notably, there is also a clear bias in this study regarding the demonstration of the SLTS mechanism in new species. Each SLe sequence in the extended database has to be related to at least one sequence in the seed database. This indicates that we will most likely not incorporate new phyla into this database unless a new methodological approach is used. In this study, we have initiated an important characterization of the SLe identity, which can be further used to unravel the presence of the SLTS mechanism in new phyla. We reported that (i) all SLe sequences have a WWG pattern at the 3′ end; (ii) a Sm binding site is always present, either in the exon or in the intron of the SL gene; (iii) the sequences are 22–25 or 36–39 nucleotides long; (iv) a SLe TTT pattern is present in almost all sequences; and (v) the RNA structure may be Y-shaped, bearing three stem-loops and a bifurcation point.

Another important contribution of this study is the observation that different SLe sequences in a given species control different transcripts and identical SLe sequences in different species control identical transcripts. We hypothesize that each SLe sequence is always related to a given set of transcripts and, therefore, the expression of the respective protein repertories could be switched on and off according to the presence of a SLe sequence. If this is correct, then the SLTS mechanism could be controlled by environmental changes and lead to translation of several processed transcripts, giving rise to a specific response. This hypothesis can be tested in further studies by activating different SLe sequences at different times and observing the phenotypic effects. Finally, we have shown that after annotation and classification of SLe-containing transcripts from all phyla that the SLTS seems to be directed to specific gene categories in each phylum.

## Author contributions

Mainá Bitar retrieved the data, designed, and conducted all of the *in silico* experiments and analyses and wrote the manuscript. Mariana Boroni participated in the data retrieval, the *in silico* experiments and in manuscript preparation. Andréa M. Macedo and Carlos R. Machado were involved in discussions, contributed with expert insights and guidance and reviewed the manuscript. Glória R. Franco designed all of the *in silico* experiments and analyses, contributed expert insights and guidance and reviewed the manuscript.

### Conflict of interest statement

The authors declare that the research was conducted in the absence of any commercial or financial relationships that could be construed as a potential conflict of interest.
