# Supplementary material for: The spliced leader trans-splicing mechanism in different organisms: molecular details and possible biological roles
Source: Front Genet. 2013 Oct 11;4:199. doi: 10.3389/fgene.2013.00199 (PMC3795323; doi:10.3389/fgene.2013.00199)
Supplement: Supplementary file 1 [file Presentation1.PDF]

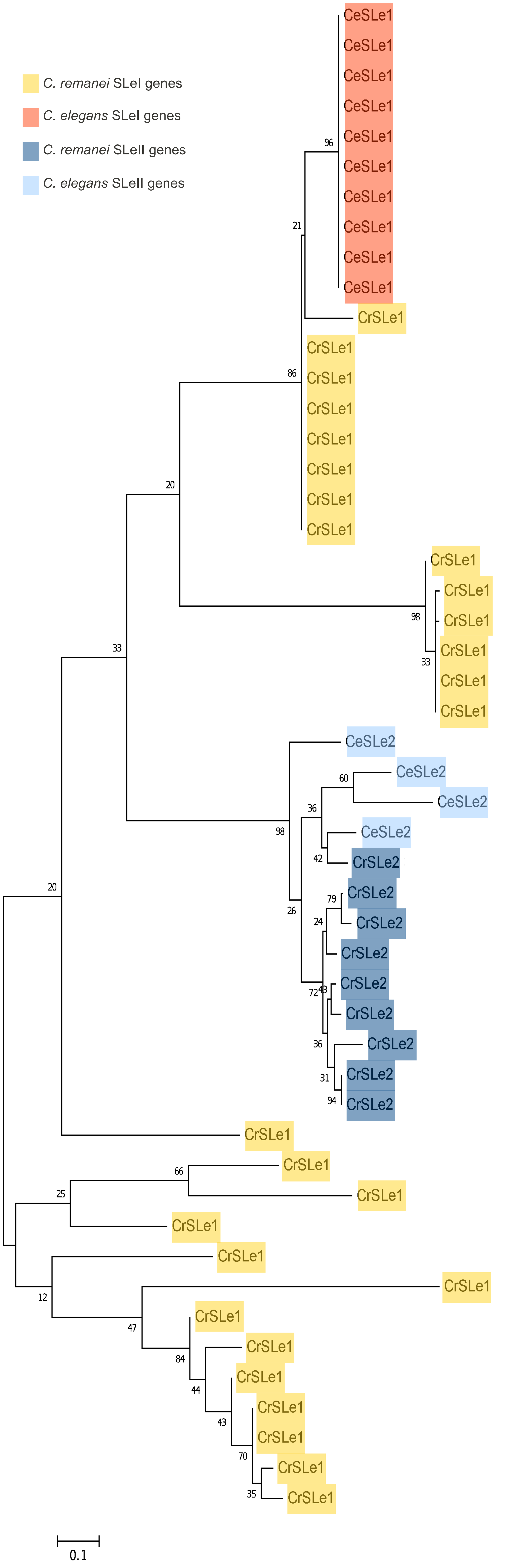

Supplementary Figure 1: Distance tree of *Caenorhabditis* SL genes. Distance tree of *C. elegans* (Ce) and *C. remanei* (Cr) SL genes containing Sm-protein binding sites showing three sequence clusters: (i) SLeI gene sequences from both species, (ii) SLeII gene sequences from both species and (iii) additional SLeI gene sequences from *C. remanei* (from top to bottom).
