# Supplementary material for: The spliced leader trans-splicing mechanism in different organisms: molecular details and possible biological roles
Source: Front Genet. 2013 Oct 11;4:199. doi: 10.3389/fgene.2013.00199 (PMC3795323; doi:10.3389/fgene.2013.00199)
Supplement: Supplementary file 2 [file DataSheet1.PDF]

SEED Database

Rotifera

GGCTTATTACAACTTACCAAGAG	*Bdelloidea sp.*
GGCTTATTACAACTTACCAAGAG	*Philodina sp.*

Chordata

GATTGGAGTATTTGGTTGTATTAAG	*Botryllus schlosseri*

Cnidaria

ACTTTTTAGTCCCTGTGTAATAAG	*Hydra vulgaris*
CAAACTTCTATTTTCTTAATAAAG	*Hydra vulgaris*

Dinoflagellate

CCGTAGCCATTTTGGCTCAAG	*Karlodinium micrum*
DCCGTAGCCATTTTGGCTCAAG	*Karlodinium veneficum*
WCCGTAGCCATTTTGGCTCAAG	*Alexandrium fundyense*
TCCGTAGCCMTTTTGGCTCAAG	*Karenia brevis*
ACCGTAGCCATCTTGGCTCAAG	*Perkinsus marinus*
CCGTAGCCATTTTGGCTCAAG	*Pfiesteria piscicida*
TCCGTAGCCATTTTGGCTCAAG	*Prorocentrum minimum*
TCCGTAGCCATTTTGGCTCAAG	*Symbiodinium sp*

Nematoda

GGGTTTAATTACCCAAGTTTGAG	*Ancylostoma caninum*
GGTTTAATTACCCAAGTTTGAG	*Angiostrongylus cantonensis*
GGGTTTAATTACCCAAGTTTGAG	*Ascaris suum*
GGTTTAATTACCCAAGTTTGAG	*Brugia malayi*
GGTTTAATTACCCAAGTTTGAG	*Caenorhabditis elegans*
GGTTTTAACCCAGTTAACCAAG	*Caenorhabditis elegans*
GGTTTAATTACCCAAGTTTGAG	*Dirofilaria immitis*
GGTTTAATTACCCAAGTTTGAG	*Globodera rostochiensis*
GGTTTAATTACCCAAGTTTGAG	*Haemonchus contortus*
GGTTTAATTACCCAAGTTTRAG	*Meloidogyne incognita*
GGTTTAATTACCCAAGTTTGAG	*Nippostrongylus brasiliensis*
GGTTTAATTACCCAAGTTTGAG	*Onchocerca cervicalis*
GGTTTRATTACCCAAGTTTGAG	*Onchocerca volvulus*
GGTTTTTTACCCAGTATCTCAAG	*Oscheius brevesophaga*

AGGTATTTACCAGATCTAAAAG	*Trichinella spiralis*
TACCGTTCAATTAATTTTGAAG	*Trichinella spiralis*
GGTTTAATTACCCAAGTTTGAG	*Trichostrongylus colubriformis*
GGTTTAATTACCCAAGTTTGAG	*Wuchereria bancrofti*

Platyhelminthe

CACCGTTAATCGGTCCTTACCTTGCAATTTTGTATG	*Echinococcus granulosus*
CACCGTTAATCGGTCCTTACCTTGCAGTTTTGTATG	*Echinococcus multilocularis*
AACCTTAACGGTTCTCTGCCCTGTATATTAGTGCATG	*Fasciola hepatica*
AACCGTCACGGTTTTACTCTTGTGATTTGTTGCATG	*Schistosoma mansoni*
CGGTCCTTACCTTGCARTTTTGTATG	*Taenia solium*

Euglenozoa

AACCAACGATTYWAAAGCTACAGTTTCTGTACTTTATTG	*Diplonema sp*
AACTAACGCTATATTTGTTACAGTTTCTGTACTWTATTGG	*Blastocrithidia culicis*
AACTAACGCTATTATTGTTACAGTTTCTGTACTTTATTGG	*Herpetomonas samuelpessoai*
AACTAACGCTATATAAGTATCAGTTTCTGTACTTTATTG	*Leishmania donovani*
AACTAACGCTATATAAGTATCAGTTTCTGTACTTTATTG	*Leishmania infantum*
CTGTACTTTATTG	*Leishmania major*
GTACTTTATTG	*Leishmania tarentolae*
AACTAACGCTATATAAGTATCAGTTTCTGTACTWTATTGG	*Leptomonas sp*
AACTAACGCTATTCTAGATACAGTTTCTGTACTTTATTG	*Phytomonas staheli*
AACTAAAGCTATTATTAGAACAGTTTCTGTACTATATTG	*Trypanosoma avium*
CGCTATTATTAGAACAGTTTCTCTATATTG	*Trypanosoma brucei*
CGCTATTATTAGAACAGTTTCTGTACTATATTG	*Trypanosoma brucei*
AACTAAAGTTATTATTGATACAGTTTCTGTACTATATTG	*Trypanosoma cobitis*
AACTAACGCTATTATTGATACAGTTTCTGTACTATATTG	*Trypanosoma conorhini*
AACGCTATTATTAGAACAGTTTCTGTACTATATTG	*Trypanosoma cruzi*
AACTAACGCTATTATTGATACAGTTTCTGTACTATATTG	*Trypanosoma cruzi*
AACTAAAGATTTTATTGTTACAGTTTCTGTACTATATTG	*Trypanosoma cyclops*
AACTAAAGTTATTATTGATACAGTTTCTGTACTATATTG	*Trypanosoma danilewskyi*
AACTAACGCTATTATTGATACAGTTTCTGTACTATATTG	*Trypanosoma dionisii*
AACGCTATTATTAGAACAGTTTCTGTACTATATTG	*Trypanosoma evansi*
AGAACAGTTTCTGTACTATATTG	*Trypanosoma gambiense*
AACTAACGCTATTATTGATACAGTTTCTGTACTATATTG	*Trypanosoma grayi*
AACTAACGCTATTATTGATACAGTTTCTGTACTATATTG	*Trypanosoma leeuwenhoeki*
AACTAACGCTATTATTGATACAGTTTCTGTACTATATTG	*Trypanosoma lewisi*
AACTAACGCTATTATTGATACAGTTTCTGTACTATATTG	*Trypanosoma mega*
AACTAACGCTATTATTGATACAGTTTCTGTACTATATTG	*Trypanosoma microti*
AACTAACGCTATTATTGATACAGTTTCTGTACTATATTG	*Trypanosoma pestanai*

AACTAACGCTATTATTGATACAGTTTCTGTACTATATTG	*Trypanosoma rangeli*
AACTAACGCTATTATTGATACAGTTTCTGTACTATATTG	*Trypanosoma rotatorium*
AACTAAAATTATTTATAATACAGTTTCTGTACTATATTG	*Trypanosoma simiae*
AACTAACGMTATTATTGATACAGTTTCTGTACTATATTG	*Trypanosoma theileri*
AACTAACGCTATTAATAGAACAGTTTCTGTACTATATTG	*Trypanosoma varani*
AACTAAAGCTTTTATTAGAACAGTTTCTGTACTATATTG	*Trypanosoma vivax*

Supplementary Table 1: The SLe SEED database.This table presents all 69 SLe sequences that comprise the SEED database and their related species and phyla.
