# Supplementary material for: The spliced leader trans-splicing mechanism in different organisms: molecular details and possible biological roles
Source: Front Genet. 2013 Oct 11;4:199. doi: 10.3389/fgene.2013.00199 (PMC3795323; doi:10.3389/fgene.2013.00199)
Supplement: Supplementary file 3 [file DataSheet2.PDF]

EXTENDED Database

Rotifera

GGCTTATTACAACTTACCAAGAG	*Bdelloidea sp.*
GGCTTATTACAACTTACCAAGAG	*Philodina sp.*
GGCTTATTACAACTTACCAAGAG	*Adineta ricciae*
**GGCTTATTACAACTTACCAAGAG**	**Consensus (3/3)**
23 nucleotides: 8A, 5C, 4G, 6T	

Chordata

TGGAGTATTTGGTTGTATTAAG	*Boltenia villosa*
GATTGGAGTATTTGGTTGTATTAAG	*Botryllus schlosseri*
ATTGGAGTATTTGGTTGTATTAAG	*Halocynthia roretzi*
GATTGGAGTCTTTGGTTGTATTAAG	*Herdmania curvata*
ATTGGAGTATTTGGTTGTATTAAG	*Polyandrocarpa misakiensis*
GAGTATTTGGTTGTATTAAG	*Styela plicata*
GATTGGAGTATTTGGTTG	*Botryllus primigenu*
**GATTGGAGTATTTGGTTGTATTAAG**	**Consensus (7/7)**
25 nucleotides: 6A, 8G, 11T	

Cnidaria

ACTTTTTAGTCCCTGTGTAATAAG	*Hydra cf.*
ACTTTTTAGTCCCTGTGTAATAAG	*Hydra littoralis*
ACTTTTTAGTCCCTGTGTAATAAG	*Hydra magnipapillata*
ACTTTTTAGTCCCTGTGTAATAAG	*Hydra oligactis*
ACTTTTTAGTCCCTGTGTAATAAG	*Hydra vulgaris*
**ACTTTTTAGTCCCTGTGTAATAAG**	**Consensus (5/7)**
24 nucleotides: 6A, 4C, 4G, 10T	
CAAACTTCTATTTTCTTAATAAAG	*Hydra magnipapillata*
CAAACTTCTATTTTCTTAATAAAG	*Hydra vulgaris*
**CAAACTTCTATTTTCTTAATAAAG**	**Consensus (2/7)**
24 nucleotides: 9A, 4C, 1G, 10T	

Dinoflagellate

TCCGTAGCCATTTTGGCTCAAG	*Alexandrium affine*
WCCGTAGCCATTTTGGCTCAAG	*Alexandrium fundyense*
WCCGTAGCCATTTTGGCTCAAG	*Amphidinium carterae*
TCCGTAGCCATTTTGGCTCAAG	*Dinophysis acuminata*
CCGTAGCCATTTTGGCTCAAG	*Dinophysis caudata*
TCCGTAGCCATTTTGGCTCAAG	*Heterocapsa rotundata*
TCCGTAGCCATTTTGGCTCAAG	*Heterocapsa triquetra*

TCCGTAGCCATTTTGGCTCAAG	*Karenia brevis*
CCGTAGCCATTTTGGCTCAAG	*Karenia mikimotoi*
CCGTAGCCATTTTGGCTCAAG	*Karlodinium micrum*
CCGTAGCCATTTTGGCTCAAG	*Karlodinium veneficum*
TCCGTAGCCATTTTGGCTCAAG	*Katodinium rotundatum*
TCCGTAGCCATTTTGGCTCAAG	*Lepidodinium chlorophorum*
CCGTAGCCATTTTGGCTCAAG	*Noctiluca scintillans*
CCGTAGCCATTTTGGCTCAAG	*Oxyrrhis marina*
TCCGTAGCCATTTTGGCTCAAG	*Peridinium foliaceum*
ACCGTAGCCATTTTGGCTCAAG	*Perkinsus chesapeaki*
ACCGTAGCCATCTTGGCTCAAG	*Perkinsus marinus*
TCCGTAGCCATTTTGGCTCAAG	*Pfiesteria piscicida*
TCCGTAGCCATTTTGGCTCAAG	*Polarella glacialis*
TCCGTAGCCATTTTGGCTCAAG	*Prorocentrum micans*
TCCGTAGCCATTTTGGCTCAAG	*Prorocentrum minimum*
TCCGTAGCCATTTTGGCTCAAG	*Symbiodinium goreaui*
**WCCGTAGCCATTTTGGCTCAAG**	**Consensus (23/23)**
22 nucleotides: 4A, 6C, 5G, 6T, 1W	

Nematoda

GGTTTAATTACCCAAGTTTGAGG	*Acrobeloides sp.*
GGTTTAATTACCCAAGTTTGAGGG	*Ancylostoma caninum*
GGTTTAATTACCCAAGTTTGAGGG	*Ancylostoma ceylanicum*
GGTTTAATTACCCAAGTTTGAGGG	*Angiostrongylus cantonensis*
GGTTTAATTACCCAAGTTTGAG	*Aphelenchus avenae*
GGTTTAATTACCCAAGTTTGAGGG	*Ascaris suum*
GGTTTAATTACCCAAGTTTGAG	*Brugia malayi*
GGTTTAATTACCCAAGTTTGAGG	*Brugia pahangi*
GGTTTAATTACCCAAGTTTGAG	*Bursaphelenchus xylophilus*
GGTTTAATTACCCAAGTTTGAGGG	*Caenorhabditis elegans*
GGTTTAATTACCCAAGTTTGAGGG	*Caenorhabditis remanei*
GGTTTAATTACCCAAGTTTGAGG	*Chandlerella quiscali*
GGTTTAATTACCCAAGTTTGAGG	*Cooperia oncophora*
GGTTTAATTACCCAAGTTTGAG	*Cylicocyclus nassatus*
GGTTTAATTACCCAAGTTTGAGGG	*Dictyocaulus filaria*
TTTAATTACCCAAGTTTGAGGG	*Dictyocaulus viviparus*
GGTTTAATTACCCAAGTTTGAG	*Dirofilaria immitis*
GGTTTAATTACCCAAGTTTGAGGG	*Ditylenchus destructor*
GGTTTAATTACCCAAGTTTGAGG	*Enterobius vermicularis*
GGTTTAATTACCCAAGTTTGAGG	*Filarial environmental*
GGTTTAATTACCCAAGTTTGAG	*Globodera rostochiensis*
GGTTTAATTACCCAAGTTTGAGG	*Haemonchus contortus*

GGTTTAATTACCCAAGTTTGAG	*Heterodera avenae*
GGTTTAATTACCCAAGTTTGAGG	*Heterodera glycines*
GGTTTAATTACCCAAGTTTGAG	*Heterorhabditis indica*
GGTTTAATTACCCAAGTTTGAGG	*Litomosoides sigmodontis*
GGTTTAATTACCCAAGTTTGAG	*Meloidogyne artiellia*
GGTTTAATTACCCAAGTTTGAG	*Necator americanus*
GGTTTAATTACCCAAGTTTGAG	*Nippostrongylus brasiliensis*
GGTTTAATTACCCAAGTTTGAG	*Oesophagostomum dentatum*
GGTTTAATTACCCAAGTTTGAGG	*Onchocerca armillata*
GGTTTAATTACCCAAGTTTGAG	*Onchocerca cervicalis*
GGTTTAATTACCCAAGTTTGAGG	*Onchocerca flexuosa*
GGTTTAATTACCCAAGTTTGAGG	*Onchocerca gibsoni*
GGTTTAATTACCCAAGTTTGAGG	*Onchocerca gutturosa*
GGTTTAATTACCCAAGTTTGAGG	*Onchocerca ochengi*
GGTTTAATTACCCAAGTTTGAGG	*Onchocerca volvulus*
GGTTTAATTACCCAAGTTTGAG	*Ostertagia ostertagi*
GGTTTAATTACCCAAGTTTGAGG	*Pristionchus pacificus*
GGTTTAATTACCCAAGTTTGAG	*Steinernema carpocapsae*
GGTTTAATTACCCAAGTTTGAG	*Steinernema feltiae*
GGTTTAATTACCCAAGTTTGAG	*Teladorsagia circumcincta*
GGTTTAATTACCCAAGTTTGAG	*Toxocara canis*
GGTTTAATTACCCAAGTTTGAG	*Trichostrongylus colubriformis*
GGTTTAATTACCCAAGTTTGAG	*Wuchereria bancrofti*
**GGTTTAATTACCCAAGTTTGAGGG**	**Consensus (45/53)**
22 nucleotides: 6A, 3C, 5G, 8T	
GGTTTAATTACCCAAGTTTAAG	*Meloidogyne incognita*
GGTTTAATTACCCAAGTTTAAG	*Meloidogyne arenaria*
**GGTTTAATTACCCAAGTTTAAG**	**Consensus (2/53)**
22 nucleotides: 7A, 3C, 4G, 8T	
GGTTTTAACCCAGTTAACCAAG	*Caenorhabditis remanei*
GGTTTTAACCCAGTTAACCAAG	*Caenorhabditis elegans*
**GGTTTTAACCCAGTTAACCAAG**	**Consensus (2/53)**
22 nucleotides: 7A, 5C, 4G, 6T	
AGGTATTTACCAGATCTAAAAG	*Trichinella spiralis*
**AGGTATTTACCAGATCTAAAAG**	**Isolated (1/53)**
22 nucleotides: 9A, 3C, 4G, 6T	
TACCGTTCAATTAATTTTGAAG	*Trichinella spiralis*
**TACCGTTCAATTAATTTTGAAG**	**Isolated (1/53)**
22 nucleotides: 7A, 3C, 3G, 9T	
GTAATAAGAAAACTCAAATAAG	*Caenorhabditis elegans*
**GTAATAAGAAAACTCAAATAAG**	**Isolated (1/53)**
22 nucleotides: 13A, 2C, 3G, 4T	
GGTTTTTACCCAGTATCTCAAG	*Oscheius brevesophaga*

**GGTTTTTACCCAGTATCTCAAG**	**Isolated (1/53)**
22 nucleotides: 5A, 5C, 4G, 8T	

Platyhelminthe

AACCGTCACGGTTTTACTCTTGTGATTTGTTGCATG	*Schistosoma haematobium*
AACCGTCACGGTTTTACTCTTGTGATTTGTTGCATG	*Schistosoma japonicum*
AACCGTCACGGTTTTACTCTTGTGATTTGTTGCATG	*Schistosoma mansoni*
**AACCGTCACGGTTTTACTCTTGTGATTTGTTGCATG**	**Consensus (3/10)**
36 nucleotides: 6A, 7C, 8G, 15T	
AACCTTAACGGTTCTCTGCCCTGTATATTAGTGCATG	*Echinostoma caproni*
AACCTTAACGGTTCTCTGCCCTGTATATTAGTGCATG	*Fasciola hepatica*
**AACCTTAACGGTTCTCTGCCCTGTATATTAGTGCATG**	**Consensus (2/10)**
37 nucleotides: 8A, 9C, 7G, 13T	
AACTATAACGGTTCTCTGCCGTGTATATTAGTGCATG	*Stephanostomum sp.*
ACGGCTCTCTGCCGTGTATATTAGTGCATG	*Clonorchis sinensis*
**AACTATAACGGYTCTCTGCCGTGTATATTAGTGCATG**	**Consensus (2/10)**
37 nucleotides: 9A, 7C, 8G, 12T, 1Y	
CACCGTTAATCGGTCCTTACCTTGCAATTTTGTATG	*Echinococcus granulosus*
CACCGTTAATCGGTCCTTACCTTGCAATTTTGTATG	*Taenia solium*
CACCGTTAATCGGTCCTTACCTTGCAGTTTTGTATG	*Echinococcus multilocularis*
**CACCGTTAATCGGTCCTTACCTTGCARTTTTGTATG**	**Consensus (3/10)**
36 nucleotides: 6A, 9C, 6G, 14T, 1R	

Euglenozoa

AACTAACGCTATATAAGTATCAGTTTCTGTACTTTATTG	*Leishmania amazonensis*
AACTAACGCTATATAAGTATCAGTTTCTGTACTTTATTG	*Leishmania braziliensis*
AACTAACGCTATATAAGTATCAGTTTCTGTACTTTATTG	*Leishmania chagasi*
AACTAACGCTATATAAGTATCAGTTTCTGTACTTTATTG	*Leishmania donovani*
AACTAACGCTATATAAGTATCAGTTTCTGTACTTTATTG	*Leishmania enriettii*
AACTAACGCTATATAAGTATCAGTTTCTGTACTTTATTG	*Leishmania guyanensis*
AACTAACGCTATATAAGTATCAGTTTCTGTACTTTATTG	*Leishmania infantum*
AACTAACGCTATATAAGTATCAGTTTCTGTACTTTATTG	*Leishmania lainsoni*
AACTAACGCTATATAAGTATCAGTTTCTGTACTTTATTG	*Leishmania major*
AACTAACGCTATATAAGTATCAGTTTCTGTACTTTATTG	*Leishmania mexicana*
AACTAACGCTATATAAGTATCAGTTTCTGTACTTTATTG	*Leishmania naiffi*
AACTAACGCTATATAAGTATCAGTTTCTGTACTTTATTG	*Leishmania panamensis*
AACTAACGCTATATAAGTATCAGTTTCTGTACTTTATTG	*Leishmania peruviana*
AACTAACGCTATATAAGTATCAGTTTCTGTACTTTATTG	*Leishmania shawi*
AACTAACGCTATATAAGTATCAGTTTCTGTACTTTATTG	*Leishmania tarentolae*
AACTAACGCTATATAAGTATCAGTTTCTGTACTTTATTG	*Leishmania tropica*
AACTAACGCTATATAAGTATCAGTTTCTGTACTWTATTG	*Leptomonas costaricensis*
AACTAACGCTATATAAGTATCAGTTTCTGTACTTTATTG	*Leptomonas seymouri*

AACTAACGCTATATAAGTATCAGTTTCTGTACTTTATTG	*Wallaceina brevicula*
AACTAACGCTATATAAGTATCAGTTTCTGTACTTTATTG	*Wallaceina inconstans*
AACTAACGCTATATAAGTATCAGTTTCTGTACTTTATTG	*Crithidia fasciculata*
**AACTAACGCTATATAAGTATCAGTTTCTGTACTTTATTG**	**Consensus (21/54)**
39 nucleotides: 12A, 6C, 5G, 16T	
AACTAACGCTATTATTGATACAGTTTCTGTACTATATTG	*Trypanosoma conorhini*
AACTAACGCTATTATTGATACAGTTTCTGTACTATATTG	*Trypanosoma cruzi*
AACTAACGCTATTATTGATACAGTTTCTGTACTATATTG	*Trypanosoma dionisii*
AACTAACGCTATTATTGATACAGTTTCTGTACTATATTG	*Trypanosoma grayi*
AACTAACGCTATTATTGATACAGTTTCTGTACTATATTG	*Trypanosoma leeuwenhoeki*
AACTAACGCTATTATTGATACAGTTTCTGTACTATATTG	*Trypanosoma lewisi*
AACTAACGCTATTATTGATACAGTTTCTGTACTATATTG	*Trypanosoma mega*
AACTAACGCTATTATTGATACAGTTTCTGTACTATATTG	*Trypanosoma microti*
AACTAACGCTATTATTGATACAGTTTCTGTACTATATTG	*Trypanosoma pestanai*
AACTAACGCTATTATTGATACAGTTTCTGTACTATATTG	*Trypanosoma rangeli*
AACTAACGCTATTATTGATACAGTTTCTGTACTATATTG	*Trypanosoma rotatorium*
AACTAACGCTATTATTGATACAGTTTCTGTACTATATTG	*Trypanosoma theileri*
**AACTAACGCTATTATTGATACAGTTTCTGTACTATATTG**	**Consensus (12/54)**
39 nucleotides: 12A, 6C, 5G, 16T	
AACTAACGCTATTAATAGAACAGTTTCTGTACTATATTG	*Trypanosoma varani*
AACTAACGCTATTATTAGAACAGTTTCTGTACTATATTG	*Trypanosoma brucei*
AACTAACGCTATTATTAGAACAGTTTCTGTACTATATTG	*Trypanosoma evansi*
AACTAACGCTATTATTAGAACAGTTTCTGTACTATATTG	*Trypanosoma cruzi*
**AACTAACGCTATTATTAGAACAGTTTCTGTACTATATTG**	**Consensus (4/54)**
39 nucleotides: 13A, 6C, 5G, 15T	
AACTAAAGTTATTATTGATACAGTTTCTGTACTATATTG	*Trypanosoma danilewskyi*
AACTAAAGTTATTATTGATACAGTTTCTGTACTATATTG	*Trypanosoma cobitis*
**AACTAAAGTTATTATTGATACAGTTTCTGTACTATATTG**	**Consensus (2/54)**
39 nucleotides: 13A, 4C, 5G, 17T	
AACTAAAGCTATTATTAGAACAGTTTCTGTACTATATTG	*Trypanosoma avium*
AACTAAAGCTTTTATTAGAACAGTTTCTGTACTATATTG	*Trypanosoma vivax*
**AACTAAAGCTWTTATTAGAACAGTTTCTGTACTATATTG**	**Consensus (2/54)**
39 nucleotides: 13A, 5C, 5G, 15T, 1W	
AACTAAAATTATTTATAATACAGTTTCTGTACTATATTG	*Trypanosoma simiae*
**AACTAAAATTATTTATAATACAGTTTCTGTACTATATTG**	**Isolated (1/54)**
39 nucleotides: 15A, 4C, 3G, 17T	
AACTAAAGATTTTATTGTTACAGTTTCTGTACTATATTG	*Trypanosoma cyclops*
**AACTAAAGATTTTATTGTTACAGTTTCTGTACTATATTG**	**Isolated (1/54)**
39 nucleotides: 12A, 4C, 5G, 18T	
AACTTACGCTATAAAAGTCACAGTTTCTGTACTTTATTG	*Trypanoplasma borreli*
AACTTACGCTATAAAAGTCACAGTTTCTGTACTTTATTG	*Cryptobia helicis*
**AACTTACGCTATAAAAGTCACAGTTTCTGTACTTTATTG**	**Consensus (2/54)**
39 nucleotides: 12A, 7C, 5G, 15T	

AACTAACGCTATTATTGTTACAGTTTCTGTACTTTATTG	*Herpetomonas mariadeanei*
AACTAACGCTATTATTGTTACAGTTTCTGTACTTTATTG	*Herpetomonas samuelpessoai*
AACTAACGCTATTATTGTTACAGTTTCTGTACTTTATTG	*Sergeia podlipaevi*
**AACTAACGCTATTATTGTTACAGTTTCTGTACTTTATTG**	**Consensus (3/54)**
39 nucleotides: 10A, 6C, 5G, 18T	
AACTAACGCTATAAAAGATACAGTTTCTGTACTTTATTG	*Bodo saltans*
AACTAACGCTAAAAAAGTTACAGTTTCTGTACTTTATTG	*Dimastigella trypaniformis*
**AACTAACGCTAWAAAAGWTACAGTTTCTGTACTTTATTG**	**Consensus (2/54)**
39 nucleotides: 13A, 6C, 5G, 13T, 2W	
AACTAACGCATTTTTTGTTACAGTTTCTGTACTTTATTG	*Blastocrithidia culicis*
**AACTAACGCATTTTTTGTTACAGTTTCTGTACTTTATTG**	**Isolated (1/54)**
39 nucleotides: 9A, 6C, 5G, 19T	
AACTAACGCTATATTTGTTACAGTTTCTGTACTWTATTG	*Blastocrithidia culicis*
**AACTAACGCTATATTTGTTACAGTTTCTGTACTWTATTG**	**Isolated (1/54)**
39 nucleotides: 10A, 6C, 5G, 17T, 1W	
AACTAACGCTATTCTAGATACAGTTTCTGTACTTTATTG	*Phytomonas serpens*
**AACTAACGCTATTCTAGATACAGTTTCTGTACTTTATTG**	**Isolated (1/54)**
39 nucleotides: 11A, 7C, 5G, 16T	
AACCAACGATTTAAAAGCTACAGTTTCTGTACTTTATTG	*Diplonema sp.*
**AACCAACGATTTAAAAGCTACAGTTTCTGTACTTTATTG**	**Isolated (1/54)**
39 nucleotides: 13A, 7C, 5G, 14T	

Supplementary Table 2: The SLe EXTENDED database. This table presents all 157 SLe sequences that comprise the EXTENDED database and their related species and phyla, as well as the nucleotide composition and generated consensus sequences with their related frequencies.
