# Supplementary material for: The spliced leader trans-splicing mechanism in different organisms: molecular details and possible biological roles
Source: Front Genet. 2013 Oct 11;4:199. doi: 10.3389/fgene.2013.00199 (PMC3795323; doi:10.3389/fgene.2013.00199)
Supplement: Supplementary file 4 [file DataSheet3.PDF]

SLe sequences not included in the SEED and EXTENDED databases

Genus	Organism name	Sequence
Apicomplexa	*Plasmodium falci parum*	(1) AATAAGAAAAATCAAATAA (2) CTTCTATTTTCTTAATAA
Arthropoda	*Bombyx mori*	TTCAATTAATTTTGAAG
Arthropoda	*Camponotus acvapi mensis*	ACTTCTATTTTCTTAAT
Arthropoda	*Copelatus sp.*	AACTAAAATTATTTATAATA
Arthropoda	*Parhyale hawaiensis*	CCTTTACCACGTTTTACTG
Arthropoda	*Pediculus humanus*	TTCAATTAATTTTGAAG
Bacteria	*Flavescence doree*	TTCAATTAATTTTGAAG
Ciliophora	*Tetrahymena thermophila*	ATGAAAAGAAAACTCAAATAAG
Echinodermata	*Strongylocentrotus purpuratus*	ATTTACCAGATCTAAAAG
Mollusca	*Chlamys farreri*	ATAAGAAAAATCAAATAA
Mycetozoa	*Dictyostelium discoideum*	GTTCAATTAATTTTGAA
Percolozoa	*Naegleria gruberi*	GTTCAATTAATTTTGAA
Phytomonas	*Phytomonas staheli*	AACTAACGCTATTCTAGATACAGTTTCTGTACTTTATTG
Plantae	*Glycine max*	GGTTTAATTACCCAAGTTTGAGGG
Plantae	*Hordeum vulgare*	GGTTTAATTACCCAAGTTTGAG
Plantae	*Oryza sativa*	ACTAACGCTAAAAAAGTAACAGTTTCTGTACTTTATTG

Supplementary table 3: This table presents putative SLe sequences that were not included in the final SEED and EXTENDED databases. The species presented in this table are members of phyla in which the SLTS mechanism was not fully described.
