# Supplementary material for: The spliced leader trans-splicing mechanism in different organisms: molecular details and possible biological roles
Source: Front Genet. 2013 Oct 11;4:199. doi: 10.3389/fgene.2013.00199 (PMC3795323; doi:10.3389/fgene.2013.00199)
Supplement: Supplementary file 5 [file DataSheet4.PDF]

TRANSCRIPTS Database

GI	Phylum	Species	Protein name
338746113	Dinoflagellate	*Lepidodinium chlorophorum*	10 kDa photosystem II polypeptide
157783490	Dinoflagellate	*Heterocapsa rotundata*	14-3-3 protein
317135000	Dinoflagellate	*Amphidinium carterae*	14-3-3 protein
334089910	Nematoda	*Heterodera glycines*	14-3-3 protein
339832116	Nematoda	*Angiostrongylus cantonensis*	14-3-3 protein
226510870	Nematoda	*Ancylostoma caninum*	14-3-3 protein
171363672	Dinoflagellate	*Perkinsus marinus*	1-deoxyxylulose-5-phosphate synthase
338746081	Dinoflagellate	*Lepidodinium chlorophorum*	1-deoxyxylulose 5-phosphate reductoisomerase
171363678	Dinoflagellate	*Perkinsus marinus*	2-C-methyl-D-erythritol 2,4-cyclodiphosphate synthase
313150329	Dinoflagellate	*Perkinsus marinus*	2-C-methyl-D-erythritol 4-phosphate cytidylyltransferase
338746095	Dinoflagellate	*Lepidodinium chlorophorum*	3,8-divinyl protochlorophyllide a 8-vinyl reductase
31980106	Platyhelminthe	*Schistosoma japonicum*	3-beta-glucuronosyltransferase
161020	Platyhelminthe	*Schistosoma mansoni*	3-hydroxy-3-methylglutaryl coenzyme A reductase
171363680	Dinoflagellate	*Perkinsus marinus*	4-hydroxy-3-methylbut-2-enyl diphosphate synthase
171363682	Dinoflagellate	*Perkinsus marinus*	4-hydroxy-3-methylbut-2-enyl diphosphate reductase
338746089	Dinoflagellate	*Lepidodinium chlorophorum*	4-diphosphocytidyl-2c-methyl-d-erythritol kinase
62362205	Rotifera	*Philodina sp*	42-9-9 protein-like
104345435	Nematoda	*Onchocerca ochengi*	5S ribosomal RNA
104345436	Nematoda	*Onchocerca armillata*	5S ribosomal RNA
104345437	Nematoda	*Onchocerca gibsoni*	5S ribosomal RNA
104345438	Nematoda	*Onchocerca flexuosa*	5S ribosomal RNA
104345439	Nematoda	*Onchocerca gutturosa*	5S ribosomal RNA
11066870	Cnidaria	*Hydra vulgaris*	5S ribosomal RNA
306850287	Nematoda	*Chandlerella quiscali*	5S ribosomal RNA (gene region)
20162463	Nematoda	*Enterobius vermicularis*	5S ribosomal RNA (gene region)
32365743	Nematoda	*Enterobius vermicularis*	5S ribosomal RNA (gene region)
158562909	Nematoda	*Filarial environmental*	5S ribosomal RNA intergenic spacer
163636559	Dinoflagellate	*Perkinsus chesapeaki*	60S ribosome subunit biogenesis-like protein
256483436	Nematoda	*Heterodera glycines*	ACC synthase-like protein
192807488	Platyhelminthe	*Fasciola hepatica*	acetate:succinate CoA-transferase
226343415	Nematoda	*Haemonchus contortus*	acetylcholine receptor monepantel-1
226343507	Nematoda	*Haemonchus contortus*	acetylcholine receptor
238801043	Nematoda	*Bursaphelenchus xylophilus*	acetylcholinesterase
33321853	Nematoda	*Meloidogyne incognita*	acetylcholinesterase
22759002	Nematoda	*Necator americanus*	acetylcholinesterase
148299221	Nematoda	*Ditylenchus destructor*	acetylcholinesterase
124021530	Nematoda	*Ascaris suum*	acidic ribosomal protein

124021548	Nematoda	*Brugia malayi*	acidic ribosomal protein P0
124021568	Nematoda	*Nippostrongylus brasiliensis*	acidic ribosomal protein P0
124021528	Nematoda	*Ascaris suum*	acidic ribosomal protein P0
163636579	Dinoflagellate	*Perkinsus marinus*	acidic ribosomal protein P1
124021622	Nematoda	*Nippostrongylus brasiliensis*	acidic ribosomal protein P1
157093184	Dinoflagellate	*Karlodinium micrum*	acidic ribosomal protein P2
317183758	Nematoda	*Haemonchus contortus*	acr-10
317183756	Nematoda	*Haemonchus contortus*	acr-15
157093368	Dinoflagellate	*Noctiluca scintillans*	actin
193890875	Dinoflagellate	*Amphidinium carterae*	actin
315451463	Dinoflagellate	*Dinophysis acuminata*	actin
315451484	Dinoflagellate	*Dinophysis caudata*	actin
224028013	Chordata	*Botryllus schlosseri*	actin
193890881	Dinoflagellate	*Amphidinium carterae*	actin (truncated)
211939907	Dinoflagellate	*Amphidinium carterae*	adenosylhomocysteinase
317134948	Dinoflagellate	*Karlodinium veneficum*	ADP ribosylation factor
317135018	Dinoflagellate	*Amphidinium carterae*	ADP ribosylation factor
27651942	Platyhelminthe	*Echinococcus granulosus*	Ag5 precursor
314907316	Nematoda	*Haemonchus contortus*	aldolase
62362201	Rotifera	*Philodina sp.*	aldolase (fba1)
11602726	Platyhelminthe	*Echinococcus multilocularis*	aldolase (fba)
163636584	Dinoflagellate	*Perkinsus marinus*	amino acid transporter-like protein
62362189	Rotifera	*Adineta ricciae*	amphiHMG1/2-like protein
60685066	Nematoda	*Caenorhabditis elegans*	anion transporter
60685088	Nematoda	*Caenorhabditis elegans*	anion transporter
269819657	Nematoda	*Ancylostoma caninum*	anticoagulant protein
223640018	Platyhelminthe	*Echinococcus multilocularis*	apolipoprotein AI binding protein
118176003	Nematoda	*Toxocara canis*	arginine kinase
15825496	Cnidaria	*Hydra vulgaris*	arginine methyltransferase
34596142	Chordata	*Herdmania curvata*	arylamine-N-transferase-like
306518491	Nematoda	*Ascaris suum*	aryl hydrocarbon receptor nuclear translocator
211939909	Dinoflagellate	*Amphidinium carterae*	ascorbate peroxidase
211939911	Dinoflagellate	*Amphidinium carterae*	aspartate carbamoyltransferase
1002681	Platyhelminthe	*Schistosoma mansoni*	ATP synthase inhibitor
193891038	Dinoflagellate	*Amphidinium carterae*	ATP synthase subunit
157783456	Dinoflagellate	*Heterocapsa triquetra*	ATP synthase subunit
300193513	Nematoda	*Ascaris suum*	ATP synthase subunit
338746083	Dinoflagellate	*Lepidodinium chlorophorum*	ATP synthase subunit
211939919	Dinoflagellate	*Karlodinium veneficum*	axoneme-like protein
34596176	Chordata	*Herdmania curvata*	BcsX1-like protein
34596150	Chordata	*Herdmania curvata*	BcsX2-like protein
157783506	Dinoflagellate	*Heterocapsa rotundata*	beta-tubulin
317134994	Dinoflagellate	*Amphidinium carterae*	beta-tubulin
125968519	Nematoda	*Necator americanus*	beta-tubulin
38154457	Cnidaria	*Hydra vulgaris*	brachyury

34596167	Chordata	*Herdmania curvata*	brain specific protein-like
13958029	Platyhelminthe	*Schistosoma mansoni*	Ca-ATPase-like protein SMA3
14029144	Cnidaria	*Hydra vulgaris*	calcyclin binding protein
157093362	Dinoflagellate	*Noctiluca scintillans*	calmodulin
157783454	Dinoflagellate	*Heterocapsa triquetra*	calmodulin
27526320	Platyhelminthe	*Echinococcus multilocularis*	calmodulin
4160166	Nematoda	*Caenorhabditis elegans*	calmodulin
339832114	Nematoda	*Angiostrongylus cantonensis*	calreticulin
995968	Nematoda	*Ascaris suum*	cAMP-dependent protein kinase catalytic subunit
665539	Nematoda	*Ancylostoma caninum*	cAMP-dependent protein kinase catalytic subunit
634054	Cnidaria	*Hydra vulgaris*	cAMP response element binding protein
190683043	Dinoflagellate	*Oxyrrhis marina*	carbonic hydrase
1002675	Platyhelminthe	*Schistosoma mansoni*	carbonyl reductase-like protein
18157497	Chordata	*Halocynthia roretzi*	cardiac troponin I
47499097	Nematoda	*Haemonchus contortus*	catalase
34596168	Chordata	*Herdmania curvata*	cathepsin L precursor-like
34596164	Chordata	*Herdmania curvata*	c-Cbl interacting protein-like
29028282	Cnidaria	*Hydra magnipapillata*	Cdc42
317135008	Dinoflagellate	*Amphidinium carterae*	centrin
171988241	Nematoda	*Pristionchus pacificus*	cGMP-dependent protein kinase
2642295	Cnidaria	*Hydra oligactis*	cGMP-dependent protein kinase
12382255	Nematoda	*Brugia malayi*	chitin synthase
211939921	Dinoflagellate	*Karlodinium veneficum*	ChlD
338746091	Dinoflagellate	*Lepidodinium chlorophorum*	chloroplast mRNA binding protein
27526314	Platyhelminthe	*Echinococcus multilocularis*	choline-o-acetyltransferase
37905664	Cnidaria	*Hydra magnipapillata*	chordin-like protein
77799300	Nematoda	*Meloidogyne arenaria*	chorismate mutase
148966143	Nematoda	*Globodera rostochiensis*	chorismate mutase
148966129	Nematoda	*Globodera rostochiensis*	chorismate mutase precursor protein
21912549	Platyhelminthe	*Taenia solium*	chromosome segregation protein
34596149	Chordata	*Herdmania curvata*	claudin-like
34596157	Chordata	*Herdmania curvata*	coagulation factor protein-like
306530922	Nematoda	*Haemonchus contortus*	collagen protein
255982731	Platyhelminthe	*Echinococcus multilocularis*	COP9 signalosome component NIP
157325493	Dinoflagellate	*Karenia brevis*	cryptochrome dash
33317339	Nematoda	*Dirofilaria immitis*	cuticlin protein
302123873	Dinoflagellate	*Perkinsus marinus*	cyclin 1
302123885	Dinoflagellate	*Perkinsus marinus*	cyclin 2
21902496	Nematoda	*Caenorhabditis elegans*	cyclin E
11602722	Platyhelminthe	*Echinococcus multilocularis*	cyclin H
2190532	Platyhelminthe	*Schistosoma mansoni*	cyclophylin-like protein
112253631	Dinoflagellate	*Prorocentrum minimum*	cyclophilin-type peptidyl-prolyl cis-trans protein (single domain)
150404781	Platyhelminthe	*Clonorchis sinensis*	cystatin-2
112253181	Dinoflagellate	*Pfiesteria piscicida*	cytochrome c

317134950	Dinoflagellate	*Karlodinium veneficum*	cytochrome c
209960871	Nematoda	*Pristionchus pacificus*	DAF-12
189303813	Nematoda	*Ancylostoma ceylanicum*	DAF-16
7381607	Nematoda	*Caenorhabditis elegans*	DAF-19
7542483	Nematoda	*Caenorhabditis elegans*	DAF-19 short variant
226437752	Nematoda	*Caenorhabditis elegans*	DEAD-box RNA helicase
21912601	Platyhelminthe	*Taenia solium*	dexyhypusine synthase
345108300	Chordata	*Botryllus schlosseri*	deleted in azoospermia-associated protein 1
325516325	Nematoda	*Haemonchus contortus*	disorganized muscle protein 1
157093196	Dinoflagellate	*Karlodinium micrum*	disulfide-isomerase-like protein
221457920	Nematoda	*Haemonchus contortus*	dopamine-gated chloride channel
298493433	Nematoda	*Heterodera avenae*	dorsal gland cell-specific expression protein
21912589	Platyhelminthe	*Taenia solium*	DSCR5 protein
112434009	Platyhelminthe	*Taenia solium*	dUTPase
193891036	Dinoflagellate	*Amphidinium carterae*	EF 1 alpha
290560678	Nematoda	Caenorhabditis elegans	elongation factor G2
114841182	Nematoda	*Ascaris suum*	elongation factor Tu1
163636573	Dinoflagellate	*Perkinsus marinus*	eIF1A
11602712	Platyhelminthe	*Echinococcus multilocularis*	eIF4A
28630932	Cnidaria	*Hydra vulgaris*	eIF4E
260401549	Nematoda	*Ascaris suum*	eIF4G
157093540	Dinoflagellate	*Oxyrrhis marina*	eIF5A
317134952	Dinoflagellate	*Karlodinium veneficum*	eIF5A
317135016	Dinoflagellate	*Amphidinium carterae*	eIF5A
14029142	Cnidaria	*Hydra vulgaris*	electron transfer flavoprotein beta subunit
5670194	Cnidaria	*Hydra vulgaris*	endothelin converting enzyme
11602718	Platyhelminthe	*Echinococcus multilocularis*	enhancer of rudimentary
21912565	Platyhelminthe	*Taenia solium*	enhancer of rudimentary
112950026	Platyhelminthe	*Echinostoma caproni*	enolase
122890449	Platyhelminthe	*Echinostoma caproni*	enolase
1839195	Platyhelminthe	*Echinostoma caproni*	enolase
1839197	Cnidaria	*Hydra cf.*	enolase
1839207	Platyhelminthe	*Stephanostomum sp.*	enolase
296940215	Nematoda	*Heterorhabditis indica*	enolase
296940217	Nematoda	*Steinernema feltiae*	enolase
301015485	Nematoda	*Haemonchus contortus*	enolase
311701369	Nematoda	*Ascaris suum*	enolase
499266	Platyhelminthe	*Fasciola hepatica*	enolase
1002615	Platyhelminthe	*Schistosoma mansoni*	enolase
27526326	Platyhelminthe	*Echinococcus multilocularis*	epidermal growth factor
338746101	Dinoflagellate	*Lepidodinium chlorophorum*	ferredoxin-NADP reductase
338746097	Dinoflagellate	*Lepidodinium chlorophorum*	ferredoxin precursor
28400470	Cnidaria	*Hydra vulgaris*	fibroblast growth factor receptor-like protein
506191	Nematoda	*Caenorhabditis elegans*	flavoprotein subunit of complex II

47078461	Cnidaria	*Hydra magnipapillata*	FLP protein
298955404	Nematoda	*Ascaris suum*	FMRFamide neuropeptide
558847	Nematoda	*Ascaris suum*	FMRFamide neuropeptide
59940164	Nematoda	*Meloidogyne incognita*	FMRFamide FLP-18 neuropeptide
50379919	Nematoda	*Teladorsagia circumcincta*	FMRFamide FLP-18 neuropeptide
50379945	Nematoda	*Dictyocaulus filaria*	FMRFamide FLP-18 neuropeptide
14029603	Nematoda	*Caenorhabditis remanei*	FOG-3
6578962	Cnidaria	*Hydra vulgaris*	frizzled receptor
338746099	Dinoflagellate	*Lepidodinium chlorophorum*	fructose-1,6 bisphosphatase
10944329	Nematoda	*Caenorhabditis elegans*	gamma-tubulin
226972858	Nematoda	*Caenorhabditis elegans*	germline defective-4
221132124	Cnidaria	*Hydra magnipapillata*	GH12687 protein
38491402	Nematoda	*Cooperia oncophora*	glutamate-gated chloride channel beta
306480896	Nematoda	*Ostertagia ostertagi*	glutamate-gated chloride channel alpha
193085055	Nematoda	*Heterodera glycines*	glutamine amidotransferase
261263055	Nematoda	*Haemonchus contortus*	glutathione peroxidase
83595138	Cnidaria	*Hydra vulgaris*	glutathione peroxidase
34596172	Chordata	*Herdmania curvata*	glutathione-requiring prostaglandin D synthase
260871343	Nematoda	*Necator americanus*	glutathione S-transferase
47717440	Nematoda	*Ancylostoma caninum*	glutathione S-transferase
34596160	Chordata	*Herdmania curvata*	glutathione S-transferase
163636581	Dinoflagellate	*Perkinsus marinus*	glutathione S-transferase
193891032	Dinoflagellate	*Amphidinium carterae*	glyceraldehyde-3-phosphate dehydrogenase (GAPDH)
168279472	Dinoflagellate	*Lepidodinium chlorophorum*	glyceraldehyde-3-phosphate dehydrogenase (GAPDH)
338746103	Dinoflagellate	*Lepidodinium chlorophorum*	glyceraldehyde-3-phosphate dehydrogenase (GAPDH)
297592030	Nematoda	*Haemonchus contortus*	glyceraldehyde-3-phosphate dehydrogenase (GAPDH)
317135010	Dinoflagellate	*Amphidinium carterae*	glyceraldehyde-3-phosphate dehydrogenase (GAPDH)
193891034	Dinoflagellate	*Amphidinium carterae*	glyceraldehyde-3-phosphate dehydrogenase (plastid GAPDH)
1002671	Platyhelminthe	*Schistosoma mansoni*	G protein beta subunit-like
62362203	Rotifera	*Philodina sp*	G protein beta polypeptide 2-like 1
310751732	Nematoda	*Ancylostoma caninum*	heat shock factor binding protein
193891024	Dinoflagellate	*Amphidinium carterae*	heat shock protein 70
238803826	Nematoda	*Meloidogyne artiellia*	heat shock protein 90
157093384	Dinoflagellate	*Noctiluca scintillans*	heat shock protein DNAJ-like
338746093	Dinoflagellate	*Lepidodinium chlorophorum*	heat shock protein DNAJ-like
312271214	Nematoda	*Angiostrongylus cantonensis*	hemoglobinase-type cysteine proteinase
11602716	Platyhelminthe	*Echinococcus multilocularis*	high mobility group-like nuclear protein 2
33465396	Platyhelminthe	*Schistosoma mansoni*	high voltage-activated calcium channel
15127837	Platyhelminthe	*Schistosoma mansoni*	high voltage-activated calcium channel
15283998	Platyhelminthe	*Schistosoma mansoni*	high voltage-activated calcium

			channel
16876478	Platyhelminthe	*Schistosoma mansoni*	histamine-responsive G-protein coupled receptor
305387464	Dinoflagellate	*Karlodinium veneficum*	histone
34596171	Chordata	*Herdmania curvata*	histone
157093380	Dinoflagellate	*Noctiluca scintillans*	histone
301298982	Dinoflagellate	*Amphidinium carterae*	histone
301298984	Dinoflagellate	*Karlodinium veneficum*	histone
301298978	Dinoflagellate	*Karlodinium veneficum*	histone deacetylase
7381168	Nematoda	*Caenorhabditis elegans*	homogentisate 1,2-dioxygenase
157093390	Dinoflagellate	*Noctiluca scintillans*	hydrolase alpha/beta superfamily
54695142	Cnidaria	*Hydra vulgaris*	hydropsin mRNA foropsin
10179028	Cnidaria	*Hydra vulgaris*	hym-323
306518489	Nematoda	*Ascaris suum*	hypoxia inducible factor 1 alpha subunit
21912569	Platyhelminthe	*Taenia solium*	HUS1 protein
22476892	Nematoda	*Caenorhabditis elegans*	inositol polyphosphate-4-phosphatase
87244349	Nematoda	*Caenorhabditis elegans*	intraflagellar transport protein variant
34596186	Chordata	*Herdmania curvata*	isopenicillin-N-epimerase-like
171188266	Nematoda	*Caenorhabditis elegans*	JUN-1C
211939923	Dinoflagellate	*Karlodinium veneficum*	ketoacyl-reductase like
55982833	Nematoda	*Caenorhabditis elegans*	kettin
146331044	Nematoda	*Steinernema carpocapsae*	LEA5 protein
302029623	Nematoda	*Ancylostoma caninum*	lectin (C-type: lectin 1)
189339129	Nematoda	*Caenorhabditis elegans*	lectin (S-type: galectin LEC-11)
298953308	Dinoflagellate	*Dinophysis acuminata*	light harvesting protein
338746085	Dinoflagellate	*Lepidodinium chlorophorum*	light harvesting protein
306530907	Nematoda	*Haemonchus contortus*	lipase
325516327	Nematoda	*Haemonchus contortus*	lipase
306530911	Nematoda	*Teladorsagia circumcincta*	lipase
34596143	Chordata	*Herdmania curvata*	lipocalin interacting membrane protein-like
157093172	Dinoflagellate	*Karlodinium micrum*	lipocalin-like protein
2895199	Chordata	*Styela plicata*	L-lactate dehydrogenase
306530900	Nematoda	*Haemonchus contortus*	LON-1 protein
306530903	Nematoda	*Teladorsagia circumcincta*	LON-1 protein
21912561	Platyhelminthe	*Taenia solium*	lysine-rich protein
2246653	Platyhelminthe	*Schistosoma mansoni*	lysophospholipase homolog
163636571	Dinoflagellate	*Perkinsus marinus*	mago-nashi-like protein
11602714	Platyhelminthe	*Echinococcus multilocularis*	mannose-6-phosphate-isomerase
293323917	Nematoda	*Acrobeloides sp.*	MAP kinase (Erk1/2)
172087828	Platyhelminthe	*Echinococcus multilocularis*	MAP kinase (MPK2 p38)
1006800	Nematoda	*Caenorhabditis elegans*	MES-3
27526318	Platyhelminthe	*Echinococcus multilocularis*	metacestode specific membrane protein
15054371	Nematoda	*Ancylostoma caninum*	metalloprotease 1 precursor
1122275	Nematoda	*HHaemonchus contortus*	microsomal aminopeptidase
21912541	Platyhelminthe	*Taenia solium*	mitotic checkpoint protein

38146934	Platyhelminthe	*Taenia solium*	mog1-like protein
596073	Nematoda	*Ascaris suum*	myoglobin
157886738	Cnidaria	*Hydra magnipapillata*	Na channel 4
289169248	Cnidaria	*Hydra magnipapillata*	Na channel 5
20269935	Nematoda	*Caenorhabditis elegans*	Na-H exchanger isoform 9a
257480820	Dinoflagellate	*Alexandrium affine*	NAP50
190612634	Dinoflagellate	*Perkinsus marinus*	natural resistance-associated macrophage protein isotype II-III
51847841	Nematoda	*Ascaris suum*	neuropeptide precursor AFP-6
157713465	Nematoda	*Haemonchus contortus*	nicotinic acetylcholine receptor
294986302	Nematoda	*Haemonchus contortus*	nicotinic acetylcholine receptor alpha subunit
294986318	Nematoda	*Teladorsagia circumcincta*	nicotinic acetylcholine receptor alpha subunit
294986332	Nematoda	*Trichostrongylus colubriformis*	nicotinic acetylcholine receptor alpha subunit
307549318	Nematoda	*Oesophagostomum dentatum*	nicotinic acetylcholine receptor alpha subunit
338736689	Nematoda	*Trichostrongylus colubriformis*	nicotinic acetylcholine receptor alpha subunit
282767684	Nematoda	*Haemonchus contortus*	nicotinic acetylcholine receptor alpha subunit
294986294	Nematoda	*Haemonchus contortus*	nicotinic acetylcholine receptor non-alpha subunit
294986310	Nematoda	*Teladorsagia circumcincta*	nicotinic acetylcholine receptor non-alpha subunit
294986326	Nematoda	*Trichostrongylus colubriformis*	nicotinic acetylcholine receptor non-alpha subunit
3560564	Cnidaria	*Hydra vulgaris*	non-receptor protein-tyrosine kinase
157703443	Cnidaria	*Hydra vulgaris*	notch
38147360	Nematoda	*Caenorhabditis elegans*	nuclear receptor NHR-100
297045078	Platyhelminthe	*Echinococcus multilocularis*	nuclear receptor NHR-1
152148437	Nematoda	*Dirofilaria immitis*	nuclear receptor NHR-6
28396037	Nematoda	*Caenorhabditis elegans*	nuclear receptor NHR-69-85-114
14133771	Nematoda	*Dirofilaria immitis*	nuclear receptor NHR-7
19919403	Nematoda	*Dirofilaria immitis*	nuclear receptor RXR
146400034	Nematoda	*Brugia malayi*	nuclear receptor RXR
163636561	Dinoflagellate	*Perkinsus chesapeaki*	nuclear transport factor
2459506	Cnidaria	*Hydra vulgaris*	nucleoporin
14029140	Cnidaria	*Hydra vulgaris*	nucleoside diphosphate kinase
21912593	Platyhelminthe	*Taenia solium*	nucleoside triphosphate kinase
193891345	Dinoflagellate	*Amphidinium carterae*	oxygen evolving complex protein
338746111	Dinoflagellate	*Lepidodinium chlorophorum*	oxygen evolving enhancer protein
338746109	Dinoflagellate	*Lepidodinium chlorophorum*	oxygen evolving protein
208972997	Chordata	*Styela plicata*	oxytocin/vasopressin-related precursor
219753665	Nematoda	*Haemonchus contortus*	P100GA2 protein
306530915	Nematoda	*Haemonchus contortus*	parasitic stage specific protein 1
306530919	Nematoda	*Teladorsagia circumcincta*	parasitic stage specific protein 1
2102725	Cnidaria	*Hydra littoralis*	Pax-A
37253912	Nematoda	*Ascaris suum*	peptide YYY and peptide PW precursor

118511753	Platyhelminthe	*Taenia solium*	peroxidase
341616325	Platyhelminthe	*Clonorchis sinensis*	peroxiredoxin
157093002	Dinoflagellate	*Perkinsus chesapeaki*	peroxiredoxin
211939925	Dinoflagellate	*Karlodinium veneficum*	pfsec61
62422147	Nematoda	*Onchocerca volvulus*	P-glycoprotein
74273593	Nematoda	*Litomosoides sigmodontis*	phosphate permease
4521174	Cnidaria	*Hydra vulgaris*	Phospholipase C
8918317	Nematoda	*Caenorhabditis elegans*	Phospholipase D
338746107	Dinoflagellate	*Lepidodinium chlorophorum*	phosphoribulokinase
1002673	Platyhelminthe	*Schistosoma mansoni*	phosphoserine phosphohydrolase-like
298954310	Dinoflagellate	*Dinophysis acuminata*	photosystem II subunit M
197267566	Chordata	*Botryllus primigenus*	Piwi
27526316	Platyhelminthe	*Echinococcus multilocularis*	pleckstrin homology domain protein 1
157093376	Dinoflagellate	*Noctiluca scintillans*	profilin-like protein
133855989	Dinoflagellate	*Katodinium rotundatum*	proliferating cell nuclear antigen
133855997	Dinoflagellate	*Peridinium foliaceum*	proliferating cell nuclear antigen
133856011	Dinoflagellate	*Prorocentrum micans*	proliferating cell nuclear antigen
133856037	Dinoflagellate	*Symbiodinium goreaui*	proliferating cell nuclear antigen
133856049	Dinoflagellate	*Alexandrium fundyense*	proliferating cell nuclear antigen
224037288	Dinoflagellate	*Peridinium foliaceum*	proliferating cell nuclear antigen
224995558	Dinoflagellate	*Karenia mikimotoi*	proliferating cell nuclear antigen
302123925	Dinoflagellate	*Perkinsus marinus*	proliferating cell nuclear antigen
217621104	Dinoflagellate	*Karenia brevis*	proliferating cell nuclear antigen
113895894	Nematoda	*Dictyocaulus viviparus*	protein disulfide isomerase 1
21912543	Platyhelminthe	*Taenia solium*	proteasome maturation protein
2073445	Cnidaria	*Hydra vulgaris*	protein kinase C
56682902	Cnidaria	*Hydra magnipapillata*	protein-tyrosine kinase
392931	Cnidaria	*Hydra vulgaris*	protein-tyrosine kinase
216296531	Cnidaria	*Hydra vulgaris*	protein-tyrosine kinase
159275	Cnidaria	*Hydra vulgaris*	protein-tyrosine kinase
27526324	Platyhelminthe	*Echinococcus multilocularis*	protoscolex specific coiled-coil protein
5019920	Nematoda	*Caenorhabditis elegans*	R151.8B protein
161367662	Platyhelminthe	*Echinococcus granulosus*	RAD9
62362185	Rotifera	*Adineta ricciae*	RAD23B
40644069	Platyhelminthe	*Echinococcus multilocularis*	Ras-related protein RAL
2706487	Cnidaria	*Hydra vulgaris*	Ras-related protein RAS-1
11602724	Platyhelminthe	*Echinococcus multilocularis*	Ras-related protein RAB4A
1619840	Nematoda	*Caenorhabditis elegans*	Ras-related protein RAB1
205364118	Cnidaria	*Hydra magnipapillata*	replication factor C
335748610	Dinoflagellate	*Karenia brevis*	replication factor C
335748684	Dinoflagellate	*Karenia brevis*	replication protein A
320000465	Nematoda	*Haemonchus contortus*	resistance to inhibitors of cholinesterase
333440756	Dinoflagellate	*Polarella glacialis*	rhodopsin
145286311	Nematoda	*Ancylostoma caninum*	ribosomal protein
157093598	Dinoflagellate	*Oxyrrhis marina*	ribosomal protein

21912545	Platyhelminthe	*Taenia solium*	ribosomal protein
317134902	Dinoflagellate	*Karlodinium veneficum*	ribosomal protein
317134954	Dinoflagellate	*Amphidinium carterae*	ribosomal protein
27526322	Platyhelminthe	*Echinococcus multilocularis*	ribosomal protein L11
112253541	Dinoflagellate	*Pfiesteria piscicida*	ribosomal protein L11
254933812	Dinoflagellate	*Karlodinium veneficum*	ribosomal protein L14-22
62362191	Rotifera	*Adineta ricciae*	ribosomal protein L18
163636577	Dinoflagellate	*Perkinsus marinus*	ribosomal protein L23
124021384	Nematoda	*Brugia malayi*	ribosomal protein L27
124021426	Nematoda	*Nippostrongylus brasiliensis*	ribosomal protein L27
310814480	Dinoflagellate	*Amphidinium carterae*	ribosomal protein L37
62362193	Rotifera	*Adineta ricciae*	ribosomal protein L37
2996184	Nematoda	*Ostertagia ostertagi*	ribosomal protein L37
62362193	Rotifera	*Adineta ricciae*	ribosomal protein L37
596081	Nematoda	*Toxocara canis*	ribosomal protein L3
269129168	Dinoflagellate	*Karlodinium micrum*	ribosomal protein S6
62362195	Rotifera	*Adineta ricciae*	ribosomal protein S7
157783458	Dinoflagellate	*Heterocapsa triquetra*	ribosomal protein S13
62362199	Rotifera	*Philodina sp*	ribosomal protein S14
112253531	Dinoflagellate	*Pfiesteria piscicida*	ribosomal protein S15
157093594	Dinoflagellate	*Oxyrrhis marina*	ribosomal protein S15
255965628	Dinoflagellate	*Pfiesteria piscicida*	ribosomal protein S24
338746115	Dinoflagellate	*Lepidodinium chlorophorum*	ribulose-bisphosphate carboxylase
163636582	Dinoflagellate	*Perkinsus marinus*	RING-H2 zinc finger protein
3283022	Nematoda	*Caenorhabditis elegans*	RNA adenosine deaminase
211939913	Dinoflagellate	*Amphidinium carterae*	RNA binding motif protein
21912583	Platyhelminthe	*Taenia solium*	RNA polymerase subunit
298570494	Dinoflagellate	*Symbiodinium sp.*	rubisco large subunit
338746117	Dinoflagellate	*Lepidodinium chlorophorum*	rubisco activase
14029154	Cnidaria	*Hydra vulgaris*	SAP domain-containing protein
306530898	Nematoda	*Haemonchus contortus*	saposin-like protein 1
284518895	Nematoda	*Ascaris suum*	scavenger decapping enzyme
27948858	Cnidaria	*Hydra vulgaris*	scythe-like protein
221119206	Cnidaria	*Hydra magnipapillata*	scythe-like protein
338746119	Dinoflagellate	*Lepidodinium chlorophorum*	sedoheptulose-1,7-bisphosphatase
34596151	Chordata	*Herdmania curvata*	separation anxiety protein-like
227955706	Nematoda	*Haemonchus contortus*	serine protease inhibitor
18699716	Nematoda	*Caenorhabditis elegans*	SID-1
42794528	Nematoda	*Caenorhabditis elegans*	SID-2
4680242	Nematoda	*Onchocerca volvulus*	SLAP-1 protein
111183468	Platyhelminthe	*Taenia solium*	SLC10
302372345	Dinoflagellate	*Perkinsus marinus*	Sm protein
317135014	Dinoflagellate	*Amphidinium carterae*	Sm protein (D1)
111034959	Platyhelminthe	*Schistosoma haematobium*	Sm protein (sl)
61967244	Nematoda	*Caenorhabditis elegans*	snRNA
86561453	Nematoda	*Caenorhabditis elegans*	snRNA

37624314	Platyhelminthe	*Clonorchis sinensis*	Superoxide dismutase (SOD)
457481	Nematoda	*Brugia pahangi*	Superoxide dismutase (Cu/Zn SOD)
83595132	Cnidaria	*Hydra vulgaris*	Superoxide dismutase (Mn SOD)
256016614	Nematoda	*Angiostrongylus cantonensis*	somatostatin receptor
11602720	Platyhelminthe	*Echinococcus multilocularis*	spliceosome-associated-protein 114
14029156	Cnidaria	*Hydra vulgaris*	SSM4-like protein
145652304	Nematoda	*Ancylostoma caninum*	START domain protein
29028284	Cnidaria	*Hydra magnipapillata*	surfeit 5 protein
324962890	Dinoflagellate	*Alexandrium fundyense*	SxtA long isoform precursor
324962888	Dinoflagellate	*Alexandrium fundyense*	SxtA short isoform precursor
1002621	Platyhelminthe	*Schistosoma mansoni*	synaptobrevin-like protein
34596155	Chordata	*Herdmania curvata*	syphon associated protein-like
146447344	Nematoda	*Caenorhabditis elegans*	SZY-20A-B
221111199	Cnidaria	*Hydra magnipapillata*	tetratricopeptide repeat domain 18
52673243	Platyhelminthe	*Echinococcus multilocularis*	TGF-beta receptor kinase 1
157093358	Dinoflagellate	*Noctiluca scintillans*	thioredoxin
29825893	Platyhelminthe	*Echinococcus granulosus*	thioredoxin glutathione reductase
1002821	Nematoda	*Brugia malayi*	thiredoxin peroxidase 1
13506760	Cnidaria	*Hydra vulgaris*	tight junction protein
21912597	Platyhelminthe	*Taenia solium*	transcription regulator
27526312	Platyhelminthe	*Echinococcus multilocularis*	transketolase
338746121	Dinoflagellate	*Lepidodinium chlorophorum*	transketolase
124021466	Nematoda	*Ascaris suum*	translationally controlled tumor protein-like protein
124697690	Nematoda	*Nippostrongylus brasiliensis*	translationally controlled tumor protein-like protein
124021490	Nematoda	*Brugia malayi*	translationally controlled tumor protein-like protein
157783494	Dinoflagellate	*Heterocapsa rotundata*	translation elongation factor-like protein
157783496	Dinoflagellate	*Heterocapsa rotundata*	translation elongation factor-like protein
1002617	Platyhelminthe	*Schistosoma mansoni*	trans-spliced mRNA
1002677	Platyhelminthe	*Schistosoma mansoni*	trans-spliced mRNA
306530892	Nematoda	*Haemonchus contortus*	transthyretin-like protein 1
306530896	Nematoda	*Teladorsagia circumcincta*	transthyretin-like protein 1
313509546	Nematoda	*Haemonchus contortus*	triose phosphate isomerase
298955314	Dinoflagellate	*Dinophysis acuminata*	triose phosphate isomerase
220897457	Platyhelminthe	*Echinococcus multilocularis*	tropomodulin
18157231	Chordata	*Halocynthia roretzi*	troponin
34596197	Chordata	*Herdmania curvata*	troponin
33590367	Nematoda	*Caenorhabditis elegans*	TRP-3 channel protein
4104016	Platyhelminthe	*Schistosoma mansoni*	tryptophan hydroxylase
157783502	Dinoflagellate	*Heterocapsa rotundata*	tubulin alpha chain
157783508	Dinoflagellate	*Heterocapsa rotundata*	tubulin folding cofactor B
148536472	Dinoflagellate	*Karenia brevis*	type I polyketide synthase-like protein
4731319	Cnidaria	*Hydra vulgaris*	tyrosine kinase receptor HTK32
780288	Cnidaria	*Hydra vulgaris*	tyrosine kinase receptor

211939917	Dinoflagellate	*Amphidinium carterae*	U2 snRNP auxiliary factor
11602706	Platyhelminthe	*Echinococcus multilocularis*	U6 snRNA-associated protein
11602710	Platyhelminthe	*Echinococcus multilocularis*	ubiquinol-cytochrome C reductase
163636563	Dinoflagellate	*Perkinsus chesapeaki*	ubiquinol-cytochrome C reductase
111610366	Platyhelminthe	*Taenia solium*	ubiquinol-cytochrome C reductase
157093352	Dinoflagellate	*Noctiluca scintillans*	ubiquitin
157783444	Dinoflagellate	*Heterocapsa triquetra*	ubiquitin
157093342	Dinoflagellate	*Noctiluca scintillans*	ubiquitin-conjugating enzyme E2
21912603	Platyhelminthe	*Taenia solium*	ubiquitin ligase
34016864	Platyhelminthe	*Echinococcus granulosus*	UDP-N-acetyl-D-galactosamine:polypeptide N-acetylgalactosaminyltransferase
38016556	Nematoda	*Haemonchus contortus*	UNC-18
290749784	Nematoda	*Oesophagostomum dentatum*	UNC-38
320000463	Nematoda	*Haemonchus contortus*	UNC-50
320000461	Nematoda	*Haemonchus contortus*	UNC-74
62362187	Rotifera	*Adineta ricciae*	unknown mRNA
11602708	Platyhelminthe	*Echinococcus multilocularis*	U-snRNP-associated cyclophilin
21703321	Chordata	*Boltenia villosa*	vacuolar ATPase
21912555	Platyhelminthe	*Taenia solium*	vacuolar ATPase membrane sector associated protein
98986201	Chordata	*Polyandrocarpa misakiensis*	vasa
10039330	Cnidaria	*Hydra vulgaris*	vasa-related protein (Cnvas2)
15042020	Nematoda	*Caenorhabditis elegans*	vha-6
211939915	Dinoflagellate	*Amphidinium carterae*	violaxanthin de-epoxidase
333440963	Nematoda	*Ascaris suum*	WAGO-1
147779783	Cnidaria	*Hydra vulgaris*	Wnt-5a protein
193208933	Nematoda	*Caenorhabditis elegans*	Y102A5D snRNA
25167991	Nematoda	*Caenorhabditis elegans*	ZK218.13 snRNA
193209308	Nematoda	*Caenorhabditis elegans*	ZK218.21 snRNA
212646679	Nematoda	*Caenorhabditis elegans*	ZK218.22 snRNA

Supplementary table 4: The TRANSCRIPTS database. This table presents all 455 SLe-containing transcripts retrieved in this study, their respective GI number, phylum, species and transcript name.
