# Supplementary material for: The spliced leader trans-splicing mechanism in different organisms: molecular details and possible biological roles
Source: Front Genet. 2013 Oct 11;4:199. doi: 10.3389/fgene.2013.00199 (PMC3795323; doi:10.3389/fgene.2013.00199)
Supplement: Supplementary file 6 [file DataSheet5.PDF]

TRANSCRIPTS Classification

**AMINO ACID METABOLISM**	**9**
Metabolism	1
Catabolism	2
Anabolism	6
**BLOOD CLOTTING**	**4**
Vessel constriction	1
Coagulation	3
**CARBOHYDRATE/ENERGY METABOLISM**	**37**
Pentose phosphate pathway	1
Anaerobic mitochondrial metabolism	3
Electron transport chain	10
Glycolisis	23
**CELL INTERACTIONS AND EXTRACELLULAR MATRIX**	**9**
Lectins	2
Tight junction proteins	2
Glycan anabolism	2
Collagen and collagenases	3
**CELL CYCLE AND APOPTOSIS**	**13**
General cell cycle regulation	5
Chromosome segregation	3
DNA damage checkpoint	3
Apoptosis	2
**CHROMATIN AND CHROMOSOME STRUCTURE**	**8**
Chromosome modeling	1
Histones	5
Other chromatin modeling proteins	2
**CYTOSKELETON AND VESICULE TRAFFIC**	**20**
Cytoskeleton components	13
Cytoskeleton organization and tunover	4
Vesicular traffic	3
**DETOXIFICATION AND STRESS RESPONSE**	**25**
Peroxides metabolism	10
Xenobiotics metabolism	6
General oxidoreductases	3
Thioredoxins	4
Response to stress	2
**DNA REPLICATION**	**12**
Replication factors	3
Proliferating cell nuclear antigen	9
**HEMOGLOBIN METABOLISM**	**3**
Heme-binding proteins	2
Hemoglobin degradation	1
**ISOPRENOIDS METABOLISM**	**8**
Isoprenoids anabolism	3
Steroids anabolism	5
**LIPID METABOLISM**	**10**
Glycolipid anabolism	1
Lipid binding	1

Lipid catabolism	8
**ORGANISMAL DEVELOPMENT**	**11**
Cell differentiation<br>Development<br>Embryogenesis<br>Regulation of life span<br>Morphogenesis	1<br>1<br>1<br>4<br>4
**MISCELLANY**	**10**
Polyketides metabolism<br>Propanoate metabolism<br>Carbonic hydrase<br>Nitrogen-binding<br>Light-sensitive protein<br>Toxins<br>Parasitism-related proteins	1<br>1<br>1<br>1<br>1<br>3<br>2
**MULTIFUNCTIONAL**	**12**
Housekeeping proteins<br>14-3-3 proteins<br>Other	4<br>5<br>3
**NEUROTRANSMISSION**	**32**
Neuropeptides and their metabolism<br>Receptors of neuropeptides	17<br>15
NO DESCRIPTION	**7**
Unknown function	7
**NUCLEOTIDE METABOLISM**	**7**
NTP/NDP balance<br>Purine metabolism<br>Pyrimidine metabolism	2<br>1<br>4
**ORGAN/TISSUE-SPECIFIC PROTEINS**	**16**
Brain proteins<br>Cuticle proteins<br>Gland proteins<br>Eggshell proteins<br>Protoscolex proteins<br>Muscle proteins	1<br>1<br>1<br>1<br>1<br>11
**PHOTOSYNTHESIS AND CARBON FIXATION**	**20**
Calvin cycle<br>Clorophyll metabolism<br>Light-responsive elements<br>Photosystem II components	8<br>2<br>5<br>5
**PROTEIN MODIFICATION**	**17**
Protein methylation<br>Protein phosphorylation	1<br>16
**PROTEIN PROCESSING AND DEGRADATION**	**13**
Proteases<br>Proteasome components<br>Ubiquitination	5<br>2<br>6
**PROTEIN PRODUCTION**	**22**
Protein complex assembly<br>Protein folding<br>Protein synthesis	1<br>9<br>12
**PROTEIN-RNA INTERACTION**	**17**

RNA binding proteins<br>snRNP components	8<br>9
**RIBOSOME COMPONENTS**	**42**
5S ribosomal RNAs<br>Acidic ribosomal proteins<br>Other ribosomal proteins	10<br>7<br>25
**RNA INTERFERENCE PATHWAY**	**4**
Argonaute protein<br>Piwi protein<br>Uptake of dsRNAs	1<br>1<br>2
**RNA TURNOVER AND PROCESSING**	**4**
mRNA turnover<br>RNA splicing	2<br>2
**SIGNALING PATHWAYS**	**26**
Calmodulins<br>Growth factors<br>Receptors<br>Ras proteins<br>Other signal transduction proteins	4<br>3<br>9<br>4<br>6
**TRANSCRIPTION**	**9**
Transcription activators<br>Transcription machinery	4<br>5
**TRANSPORT**	**27**
Ion transporters<br>Molecular pumps<br>Nuclear transporters<br>Small molecules/solute transporters<br>Biomolecules transporters<br>Other transporters	11<br>3<br>3<br>4<br>4<br>2

Supplementary table 5: The TRANSCRIPTS classification. This table presents all 30 classes and their subclasses in which SLe-containing transcripts retrieved in this study were classified according to their main biological functions.
